# Melanoma Cells Produce Large Vesicular-Bodies That Cause Rapid Disruption of Brain Endothelial Barrier-Integrity and Disassembly of Junctional Proteins

**DOI:** 10.3390/ijms24076082

**Published:** 2023-03-23

**Authors:** Dayna Spurling, Akshata Anchan, James Hucklesby, Graeme Finlay, Catherine E. Angel, E. Scott Graham

**Affiliations:** 1 Department of Molecular Medicine and Pathology, School of Medical Sciences, Faculty of Medical and Health Sciences, University of Auckland, Auckland 1023, New Zealand; 2 Centre for Brain Research, University of Auckland, Auckland 1023, New Zealand; 3 School of Biological Sciences, Faculty of Science, University of Auckland, Auckland 1010, New Zealand; 4 Maurice Wilkins Centre, University of Auckland, Auckland 1010, New Zealand

**Keywords:** brain endothelial cells, melanoma, brain metastasis, ECIS, oncosomes, apoptotic bodies, vesicles

## Abstract

It is known that many cells produce extracellular vesicles, and this includes a range of different cancer cell types. Here we demonstrate the profound effects of large vesicular-like bodies produced by melanoma cells on the barrier integrity of human brain endothelial cells. These vesicular-bodies have not been fully characterised but range in size from ~500 nm to >10 µm, are surrounded by membrane and are enzymatically active based on cell-tracker incorporation. Their size is consistent with previously reported large oncosomes and apoptotic bodies. We demonstrate that these melanoma-derived vesicular-bodies rapidly affect brain endothelial barrier integrity, measured using ECIS biosensor technology, where the disruption is evident within ~60 min. This disruption involves acquisition of the vesicles through transcellular uptake into the endothelial cells. We also observed extensive actin-rearrangement, actin removal from the paracellular boundary of the endothelial cells and envelopment of the vesicular-bodies by actin. This was concordant with widespread changes in CD144 localisation, which was consistent with the loss of junctional strength. High-resolution confocal imaging revealed proximity of the melanoma vesicular-bodies juxtaposed to the endothelial nucleus, often containing fragmented DNA themselves, raising speculation over this association and potential delivery of nuclear material into the brain endothelial cells. The disruption of the endothelial cells occurs in a manner that is faster and completely distinct to that of invasion by intact melanoma cells. Given the clinical observation of large vesicles in the circulation of melanoma patients by others, we hypothesize their involvement in weakening or priming the brain vasculature for melanoma invasion.

## 1. Introduction

### 1.1. Melanoma Brain Metastasis

Melanoma is the most aggressive skin cancer, resulting in 80% of all skin cancer-related deaths [1,2]. The most popular locations for metastasis include secondary skin regions, extra-regional lymph nodes, lungs, liver, the gastrointestinal tract and the central nervous systems (CNS) [3,4]. Melanoma metastasis to the CNS, however, has significantly worse prognosis compared to other locations, commonly associated with the terminal stage of the disease [5,6,7]. In order for malignant cells to invade the CNS parenchyma, they must pass the blood–brain barrier (BBB) [8,9].

The BBB defines the distinct CNS microvasculature consisting of multiple cellular components including highly specialised endothelial cells, astrocytes and pericytes, which together form the neurovascular unit (NVU) [10,11]. The CNS is comprised of continuous non-fenestrated vessels, where paracellular permeability is regulated with a range of inter-endothelial junctions, present at high density at the cerebral endothelium [7,8]. Endothelial tight junctions (TJs) form junctions closest to the apical face of the paracellular cleft, maintained by proteins Claudins (−5, −12), Occludins, junctional adhesion molecules (JAMs) and the cytoplasmic anchoring proteins Zonula Occludens (ZO-1, ZO-2) [12]. Additionally, basolateral adherens junctions (AJs) play a role in regulating barrier function and maintaining cell morphology. VE-Cadherin, or CD144, is a cadherin characterised in the intercellular connections of cerebral vascular endothelial cells and sustains the cell structure through its link to the actin cytoskeleton [13]. Additionally, CD144 is highly localised to the basolateral cell borders and junctions, and therefore any resulting disruption of TJs typically results in a corresponding loss of CD144, highlighting its importance in the integrity and structure of the BBB [14]. AJs also contain platelet endothelial cell adhesion molecules (PECAM) that are similarly involved in endothelial junction maintenance. The ability of the BBB to regulate CNS homeostasis is essential for proper neuronal function, while also protecting exposure of the CNS to pathogens, inflammation and disease [10].

While the BBB works to prevent many cancers from entering the brain, some cancers have adapted and developed mechanisms in order to penetrate the protective layer. In vivo imaging reveals melanoma cells slowing down and interacting with the brain microvasculature, as they adhere to branch points allowing for extravasation through the tight cerebral endothelial cell junctions [15]. Furthermore, there is evidence that melanoma cells upregulate cell adhesion molecules such as VLA-4 that allows binding to the endothelial vascular cell adhesion molecule, VCAM-1, aiding melanoma extravasation [16].

The prevalence of brain metastasis in melanoma patients is high, with around 50–75% of patients developing CNS involvement once the disease has become metastatic [2,17,18]. The number of brain metastatic lesions in melanoma patients observed in post-mortem brains, however, has been noted to be up to 90% [19]. Furthermore, CNS metastases have differential clinical staging and management to any other metastatic site, reflective of the severity of this form of the disease [3]. Therefore, the need to find new treatments for brain metastasis is a large area of interest in cancer therapy. Even with the use of different therapies to treat brain metastasis, several studies report a median overall survival of approximately 4 months [20,21]. Currently all forms of brain metastasis therapies are targeted to treating the tumours once they have already formed [2]. However, due to the short time frame patients have from brain metastasis diagnosis, targeting the metastatic cells whilst they are in the bloodstream is a more attractive option than treating the developed secondary brain tumour. In order to target brain metastasis prior to formation, treatment would need to target the specific mechanisms the melanoma cells use to invade the BBB [22]. It is therefore of the utmost importance to understand and characterise the interactions between melanoma cells and cerebral endothelial cells to prevent melanoma brain metastasis. Understanding these mechanisms is an ever-evolving field of research, which in more recent years has shown evidence of tumour cell-derived extracellular vesicles displaying an increasingly prominent role.

### 1.2. Extracellular Vesicles in Cancer Progression and Metastasis

Extracellular vesicles (EVs) are membrane-bound particles produced essentially by all cell types, involved in a complex and highly conserved intercellular communication system [23]. EVs are becoming relevant in many research fields due to their ability to package and exchange proteins, lipids and nucleic acids. The function of EVs as “messengers” in tumour progression and metastasis has emerged as a key mechanism employed by many cancers [24,25,26,27]. Current evidence suggests that the rate of vesicle release is enhanced through oncogenic processes, resulting in densely packed vesicles not seen under normal physiological circumstances [28,29,30]. Additionally, EVs have been shown to travel to distant sites in vivo, evidently crucial to affecting metastasis [31,32,33,34].

“Extracellular vesicles” is an umbrella term for a plethora of secreted circulating vesicles that can be characterised in relation to their size, secretion pathway and tissue origin. Tumour-derived vesicles can be divided into the categories of smaller EVs, from exosomes and nanovesicles to microvesicles, and larger EVs, including large oncosomes and apoptotic bodies [35,36]. Several studies have been conducted with the smaller EV population, predominantly focusing on exosomes and nanovesicles. For example, breast cancer studies have shown that tumour-derived EVs promote brain metastasis through BBB disruption [32,37]. Morad et al. [38] have shown that breast cancer-derived EVs can breach an intact BBB in vivo through a transcytosis mechanism. Cancer cells utilise the release of EVs to deliver oncogenic proteins and molecules to the brain endothelium directly or result in the dissolution of key structural molecules of the endothelial cells in order to aid penetration of the BBB [39]. Evidently, of the defined types of vesicles secreted from brain metastatic tumours, exosomes and microvesicles have more defined roles in BBB breakdown [40]. Metastatic melanoma is also documented to secrete pro-tumorigenic EVs in large quantities. Peinado, Alečković [16] showed that exosomes derived from a metastatic melanoma cell line induce vascular leakiness at the pre-metastatic site. Though these melanoma EVs also increased metastasis to various organs including the brain, their role in BBB function has yet to be elucidated.

Classically, EVs have been described as smaller, nanoparticle-sized vesicles, not typically reaching larger than 1 µm, unless described in an apoptotic setting [36]. However, the more recent identification of a much larger class of EV, ranging in size from 1 to 10 µm called large oncosomes (LO) have been implicated in an oncogenic setting [41,42]. Large oncosomes are only present in the case of aggressive cancers and do not exist under normal physiological cell–cell communication [42,43]. Additionally, large oncosomes have been documented to be present in the circulation of prostate cancer patients. Their presence in circulation has been seen to discriminate those with metastatic disease from those with early stage, confined prostate cancer [42]. However, in the context of melanoma brain metastasis, the mechanisms by which tumour-derived large EVs interact with and damage the restrictive BBB is still unknown.

Apoptotic bodies (ApoBDs) are another population of large EVs formed during the programmed cell death response. They are created by all human cell types in the homeostatic mechanism of apoptosis [44,45]. The role of ApoBDs in cancer metastasis and potential BBB breakdown is the least defined in the current literature, and in addition to this, ApoBDs often remain uncharacterised after systematic removal from studies analysing the effects of EVs [46]. Yet apoptosis is an extremely relevant process in tumorigenesis and metastasis. The majority of tumour cells that enter the circulation do not survive due to the activation of cell death machinery through various stressors. These include (i) the loss of cell–cell contacts; (ii) the lack of growth factors essential for survival provided by the tumour microenvironment; and (iii) recognition and destruction by the immune system, all of which lead to resultant programmed cell death including anoikis, apoptosis, necroptosis or autophagy [47,48,49]. Therefore, cancer cell metastasis is an example of an important physiologically relevant setting in which ApoBDs are present. The literature in the space of apoptotic circulating tumour cells (aCTCs) and their corresponding debris (ApoBDs) shows evidence of a pro-metastatic role emerging, although historically aCTCs have been described as a marker for response to chemotherapy [50,51,52]. Intriguingly, studies note large numbers of **apoptotic** CTCs in circulation, more so than the number of **viable** CTCs [52,53]. While their involvement in metastasis is not understood, the hypothesis that these vesicles could be involved in metastasis and priming premetastatic niches could be devastating [51,54]. The evidence of large oncosomes, apoptotic bodies and other tumour-derived EVs in the circulation of patients with metastatic cancer is undeniable, and the correlation to increased metastatic potential albeit significant requires further investigation.

### 1.3. New Zealand Melanoma and Their Vesicular-Bodies

We have previously shown that patient-derived metastatic melanoma lines induce rapid disruption of human brain endothelial cells, specifically by adhering at the paracellular junctions [55]. These results are in line with the current literature on this topic [22,56,57]. Analysis of melanoma media components revealed 15 highly concentrated secreted proteins, of which TGFβ and ANGPTL-4 showed little evidence of affecting endothelial barrier integrity [58]. Through this previous work, we discovered a sub-population of melanoma cell material released by the patient-derived melanoma lines. Together, these results proposed that melanoma lines may in fact release other entities that resemble melanoma-derived large vesicles. Importantly, these entities appeared to be produced in abundance by the adherent cells, indicating an inherent function for disease progression, rather than the incidental release of dead cells. Currently, the involvement of large EVs or ApoBDs in melanoma metastasis has yet to be elucidated. While it is estimated that up to one million melanoma cells per gram of tissue are shed from the primary tumour every day, the number of viable melanoma cells in the circulation is estimated to be as low as 1 melanoma cell per 5 billion blood cells [49,59,60,61]. Yet melanoma cells are deemed to have one of the highest affinities for metastasis of any other cancer, with the ability to colonise a range of secondary tissues. This suggests that melanoma cells can employ a variety of mechanisms in addition to the upregulation of adhesion molecules and secretory metastatic factors. It is likely that the efficiency of these cells in disrupting the BBB is the result of a multitude of mechanisms working synergistically to do so, resulting in many different avenues of investigation being undertaken.

The primary goal of this paper was to ascertain whether the melanoma-derived particles observed in the culture affected the barrier integrity of human brain endothelial cells. We hypothesised that the particles would have a detrimental effect on the brain endothelial barrier, and the mechanism of this would differ from the effects of the melanoma cells proper. It was logical therefore to pursue the functional relevance of the suspension material first, prior to conducting a comprehensive analysis of its identity. Comprehensive molecular profiling was an attractive avenue. However, the time required, resources available and cost-prohibitive nature for completing this profiling was not justified prior to finding evidence of functional relevance of the vesicular-bodies.

## 2. Results

### 2.1. Initial Detection of the NZM Suspension Subpopulation Shows Clear Evidence of Enzymatic Activity

During the culture of different patient-derived melanoma lines (NZM lines), it was noted that certain lines produced low levels of suspension cells after several days in culture. This observation was particularly evident with the NZM7 melanoma cultures compared with other NZM lines, −48 and −74, where the subpopulation was relatively abundant after about ~7 days in culture (Supplemental Figures S2 and S3). Our initial thoughts were that these were dead or dying cells, or metastatic cells becoming less adherent. Given our previous work investigating melanoma cell adhesion and disruption of the brain endothelial barrier integrity [55,58], we were particularly intrigued as to whether the NZM suspension populations had similar biological activity. This was an interesting consideration, especially if it proved that the suspension population had a more metastatic phenotype. For simplicity, this population is initially referred to as NZM7-S for our initial experiments.

**Figure 1:**
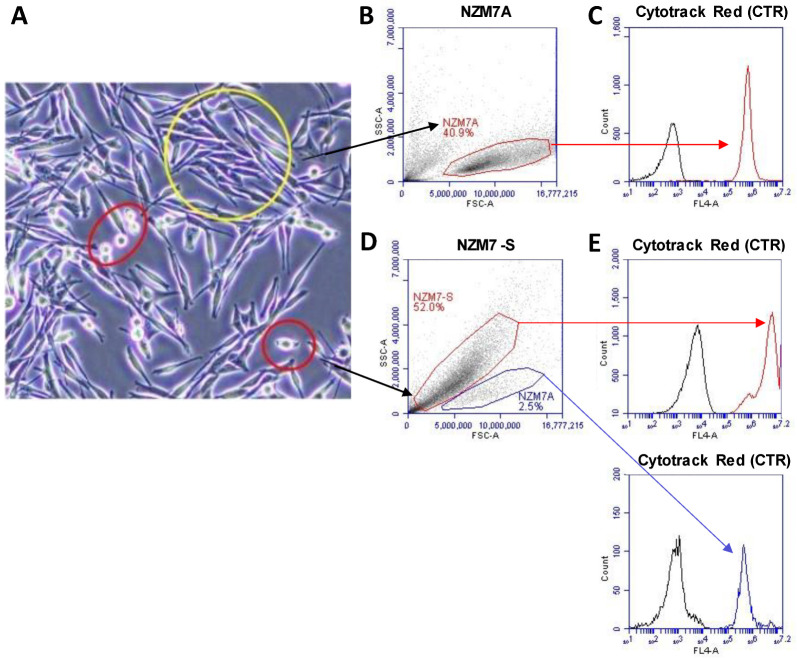
NZM7 imaging and gating strategies. (**A**) NZM7 cell line in culture. Yellow circle shows NZM7A, red circles show NZM7-S. (**B**,**D**) Scatter profile of gated NZM7A and NZM7-S according to density in *y*-axis (SSC-A) and size in *x*-axis (FSC-A). (**C**,**E-top**) CTR fluorescence (red) of the suspension population compared to the cells autofluorescence (black) as measured in channel FL4-672/25 filter. (**E-bottom**) CTR fluorescence (blue) of the NZM7A population in the NZM7-S collection. Data show 1 representative experiment, which is from 3 independent experiments.

Figure 1 shows phase contrast images of an NZM7 culture after 1 day in culture. Most of the cells were stellate and adherent; however, there were a few cells with a more phase bright appearance (circled in red). Figure 1B shows the forward and side scatter profile of live NZM7 cells and their ability to incorporate CytoTrack Red (CTR) (Figure 1C). This showed the homogeneous incorporation of CTR into the living melanoma cells and its suitability for assessing the cells downstream by flow cytometry. In comparison, the forward and side scatter profiles of the suspension population were very different. The forward scatter (FSC) was less, but the side scatter (SSC) was much greater. This is unusual and suggests the population was smaller in diameter but also considerably denser (more compact) (Figure 1D,E). The suspension population also had a higher auto-fluorescence (Figure 1E), consistent with being denser material, and it also labelled incredibly efficiently with the CTR stain. CTR only becomes fluorescent after diffusion across the cell membrane and incorporation into intracellular proteins by esterases following removal of the fluorescence blocker. This implies that the NZM7-S material is surrounded by membrane and contains active esterases. Of note is the bi-modal CTR staining distribution indicative of differences in the staining efficiency or CTR retention by the NZM7-S population. This profile was **not** consistent with the adherent cells but did indicate that the NZM7-S population is highly enzymatically active and enveloped in the cell membrane. Notably, Figure 1D shows a second scatter profile that resembles the adherent population, suggesting that our collected suspension material included some of the parent line as well. This could be cells that were in suspension and dividing during the time of collection. 

### 2.2. NZM Suspension Subpopulation Substantially and Rapidly Decreases Brain Endothelial Barrier Resistance

**Figure 2:**
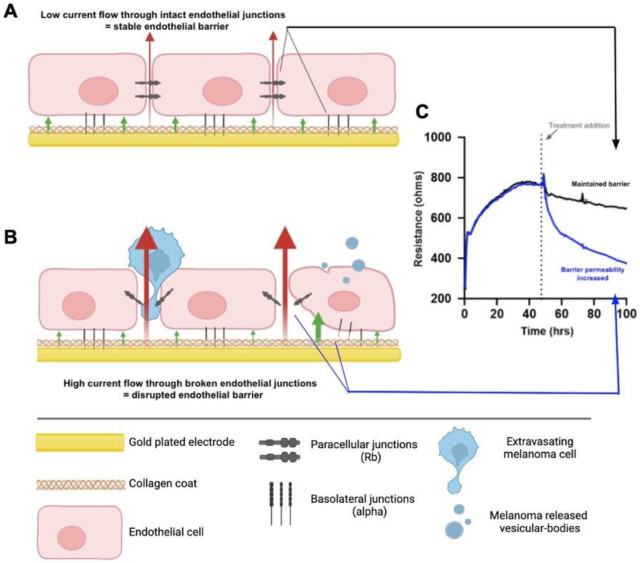
Schematic explaining ECIS theory. (**A**) Endothelial cells grown on the gold-plated electrodes of the ECIS plate that are coated with collagen, forming a confluent monolayer. The monolayer is formed via intercellular junctions and junctions with the collagen substrate, creating the paracellular and basolateral junctions, respectively. The formation of these junctions prevents high current flow from the electrode through the cells, where the red and green arrows represent the paracellular (Rb) and basolateral (alpha) current flow, respectively. This maintained electrical resistance is shown in graph C (Black). (**B**) The endothelial monolayer following treatment with factors that disrupt the endothelial barrier, such as melanoma cells or melanoma vesicular-bodies; the junctions weaken, allowing for greater current flow through the cells. This results in a decreased electrical resistance, shown in graph (**C**) (Blue). (**C**) A model ECIS graph depicting the plateau in resistance formed prior to treatment, indicating a fully formed endothelial barrier, and the corresponding results from 1. No treatment added (Black) and 2. Melanoma cell addition (Blue). Created with BioRender.com by Dayna Spurling; Reprinted/adapted with permission from Ref. [55]. 2019, Anchan, A.

**Figure 3:**
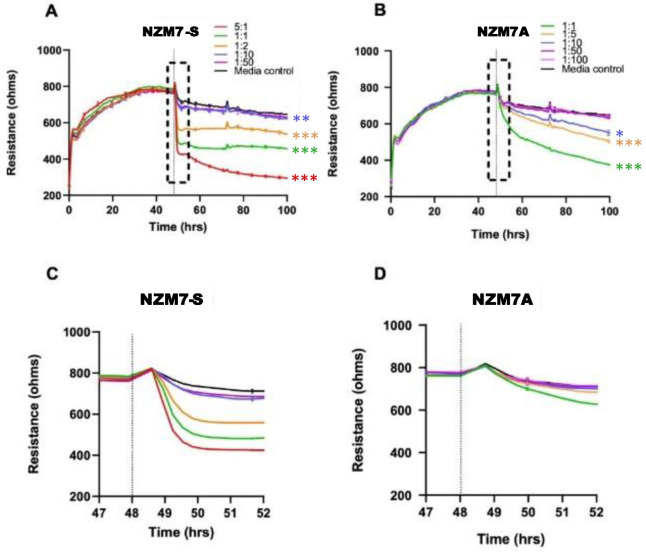
Melanoma-mediated decrease in brain endothelial barrier resistance. Graphs show unmodelled resistance (at 4000 Hz) of hCMVECs over time after the addition of the NZM7-S and NZM7A cell lines (**A**,**B**, respectively). hCMVECs were seeded at 20,000 cells per well. NZM7 cell lines were added at different Effector:Target (E:T) ratios, where 1:1 shows 1 NZM7 cell added to 1 endothelial cell. Graphs (**C**,**D**) are representative of the 4 h time period within the box of respective graphs (**A**,**B**). Data show the mean ± SD (*n* = 3 wells) from 1 experiment, which is representative of 3 independent experiments. The 60 h endpoints from independent experiments were compared relative to their media control using two-way ANOVA with Tukey’s range test (* *p* < 0.05, ** *p* < 0.01, *** *p* < 0.001).

Next, we assessed whether there was any effect of the NZM suspension population on the barrier integrity of the brain endothelial cells (hCMVECs). This was conducted using ECIS technology, which we have used extensively [55,58,62,63,64,65], utilising its powerful barrier modelling capabilities [55,64,66]. Figure 2 shows a schematic diagram, explaining the ECIS technology. In the first series of endothelial barrier experiments, the NZM7 cells were grown for approximately 7–8 days in a T75 flask to generate a large yield of NZM suspension material (see Supplemental Figure S2). These were harvested and added to brain endothelial cells (hCMVECs) that had formed a stable barrier (Figure 3).

**Figure 4:**
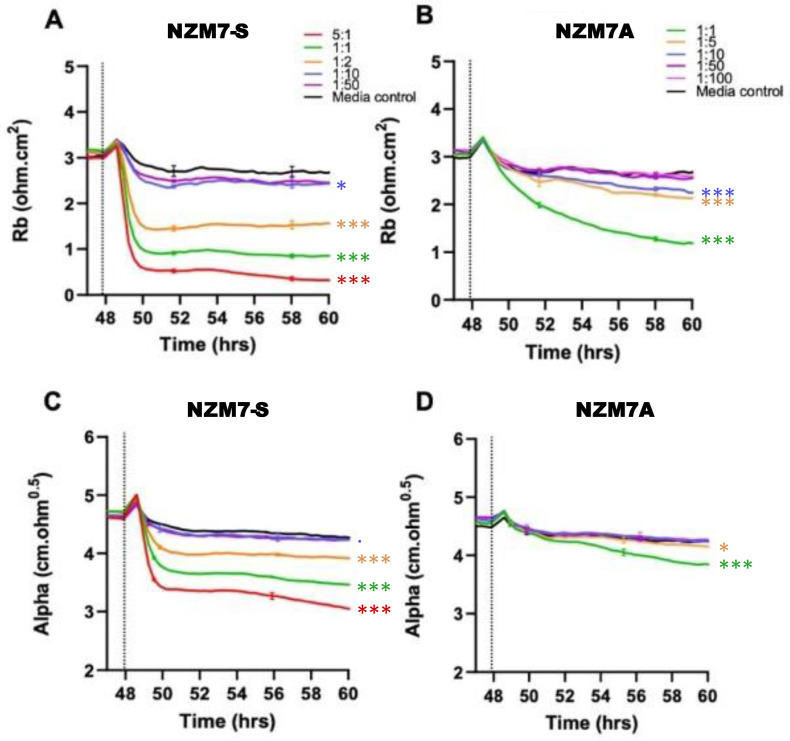
Melanoma-mediated disruption of the brain endothelium. Graphs (**A**,**B**) show modelled paracellular resistance (Rb) of hCMVECs after the addition of NZM7-S and NZM7A cell types, respectively. Graphs (**C**,**D**) show modelled basolateral resistance (Alpha) of hCMVECs after the addition of NZM7-S and NZM7A cell types, respectively. NZM7 cells were added at 48 h. The data show the mean ± SD (*n* = 3 wells) from 1 experiment, which is representative of 4 independent experiments. The 60 h endpoints from independent experiments were compared relative to their media control using two-way ANOVA with Tukey’s range test (*p* < 0.1, * *p* < 0.05, *** *p* < 0.001).

The NZM7-S population was added at a range of E:T ratios to assess sensitivity of the endothelial cells. The effect of the NZM7-S population was instantaneous and substantial (Figure 3 and Figure 4), resulting in loss of endothelial adhesion, which was evident for E:T ratios greater than 1:10. This was considerably faster and mediated a greater effect than the corresponding adherent melanoma cells (Figure 3B,D). This suggested that the NZM7-S population was more potent at affecting the endothelial integrity than the intact melanoma cells.

Figure 4 shows the modelled data from Figure 3, where Rb represents the barrier properties in the paracellular space, and Alpha represents the basolateral adhesion of the endothelial cells (see Figure 2 highlighting these locations and [55,58,67] for detailed explanations of ECIS modelling). It is clear that the effect of the NZM7-S material was much faster than that of the living melanoma cells (NZM7A), where loss of Rb was considerable within 1–2 h of NZM7-S addition.

Equally intriguing is that the modelled Alpha was changing in the exact same time-frame as Rb following addition of the NZM7-S population. However, following addition of the parent NZM7 melanoma cells, the loss of basolateral adhesion of the hCMVECs occurred several hours after changes in Rb. The delay in the change to Alpha is an observation we have reported previously [55]. This delay occurs because the melanoma cells target the paracellular junctions initially and then migrate through the paracellular space and junctions, forcing the endothelial cells apart. There is a delayed but sustained loss of basolateral adhesion of the brain endothelial cells, as it takes several hours for the melanoma cells to remove the junctional molecules and push through the monolayer [55]. As the change to Alpha following NZM7-S addition is instantaneous and simultaneous with Rb, it implies that the mechanism of barrier disruption is completely different. This suggests that the compartments of the paracellular barrier and the basolateral adhesion of the endothelial cells are affected in the same timeframe, indicating a substantial and critical impact by the NZM7-S population. Note in the previous Figure 1D we observed some NZM7A in our NZM7-S population. Hence, at this point, we suggest that it is a concoction of some adherent NZM7A with the suspension material that caused the deleterious effect on both the paracellular and basolateral compartments of the barrier. However, as evident, this effect was faster and larger than what we saw upon adding the NZM7A alone [55,66].

Culturing other melanoma lines (NZM48, NZM41 and NZM98) showed a similar effect, where the adherent cells took longer to disrupt the endothelial barrier compared to their suspension material (Supplemental Figure S4). Even at the lower titrations of 10% (~E:T of 1:1), the NZM-suspension particles were more deleterious to the barrier in the first two hours of treatment compared to their cancer cells proper (also added at an E:T of 1:1). Such a critical event would be consistent with the NZM7-S population causing the death or substantial structural changes to the cytoskeletal structure of the endothelial cells.

### 2.3. Imaging Data Show NZM7-S Population Adheres to and Disrupts Brain Endothelial Barrier

**Figure 5:**
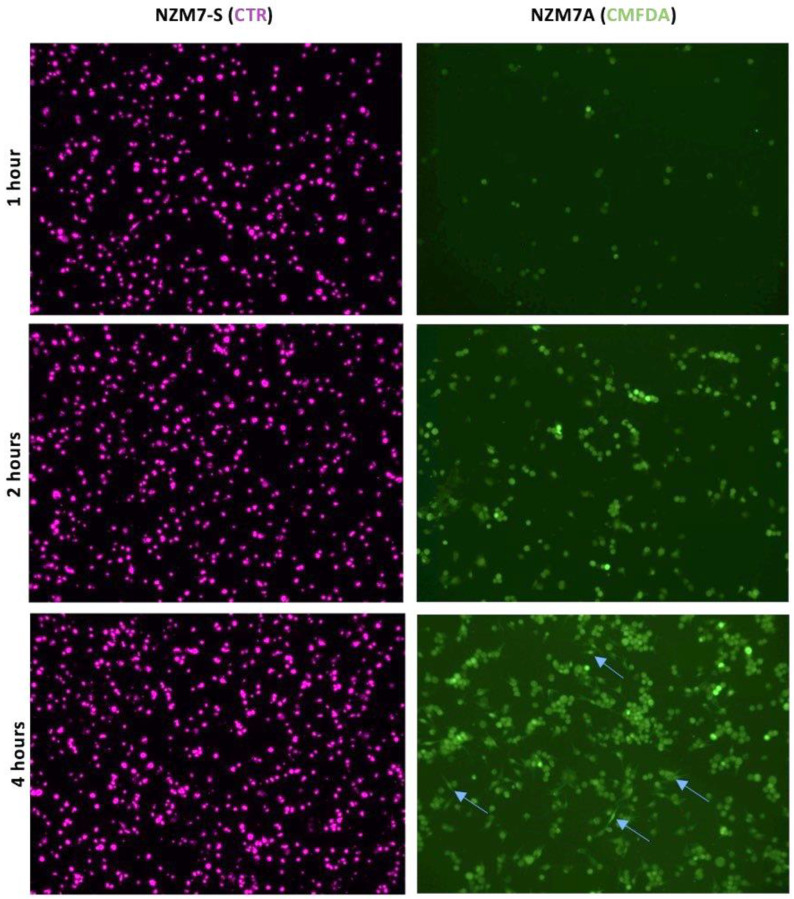
NZM7-S adhere to human cerebral microvascular endothelial cells at a faster rate than NZM7A cells. hCMVECs were seeded at 20,000 cells per well in three identical plates. NZM7-S and NZM7A cells were stained with live cell stains CTR and CMFDA, respectively. After the formation of a confluent monolayer of hCMVEC (endothelial cells) at approximately 48 h, the melanoma cells were added to the apical face of hCMVECs at an E:T ratio of 1:1. Blue arrows show evidence of NZM7A cells elongating at 4 h. The presence of the hCMVECs is not revealed in the respective images. Melanoma populations were co-cultured on top of the hCMVECs for 1, 2 and 4 h, at which time points non-adherent material was removed and cells fixed in PFA. Images were acquired at 10× on an EVOS microscope.

Next, we conducted a series of imaging-based studies to better understand how the NZM7-S population was affecting the brain endothelial cells. Firstly, we assessed the adhesion of melanoma cells to the endothelial monolayer (Figure 5) and observed that CTR-stained NZM7-S attached immediately to the endothelial monolayer in large numbers compared to the NZM7A cells (green). The CTR-stained NZM7-S population adhered abundantly in the first hour of their addition, whereas the NZM7A cells (green) clearly increased adhesion over time. Some parent NZM7A cells also showed elongation at 4 h, which we have demonstrated previously as a characteristic of intercalation within the endothelial monolayer (blue arrows). For clarity of the images in Figure 5, the hCMVEC endothelial cells were not visualised, but they do represent a monolayer of cells.

**Figure 6:**
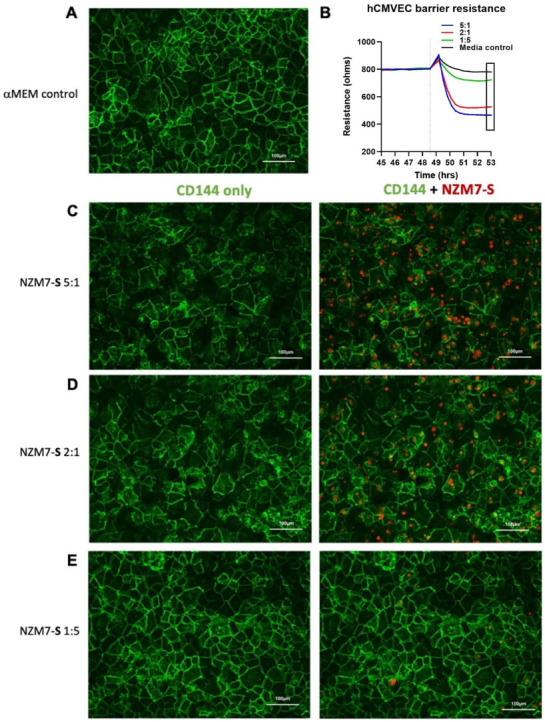
Human cerebral microvascular endothelial cell CD144 staining after NZM7-S treatment at varied concentrations. hCMVECs were seeded at 20,000 cells per well. NZM7-S were live stained with CytoTrack Red (CTR; red in image) and applied to the apical face of a confluent endothelial monolayer. Cells were co-cultured for 4 h, washed and then fixed. Junctions were stained with anti-CD144 (green). (**A**) An image from the media control well. (**B**) A paired ECIS experiment, where the boxed region shows the point of fixing for the ICC images (4 h). (**C**,**D,E**) The images of the melanoma-treated wells at different E:T ratios, noted on the left side of the panel. Images were acquired on the Operetta CLS imaging system. Full view images scale bar = 100 µm. Images are representative of 2 independent experiments.

**Figure 7:**
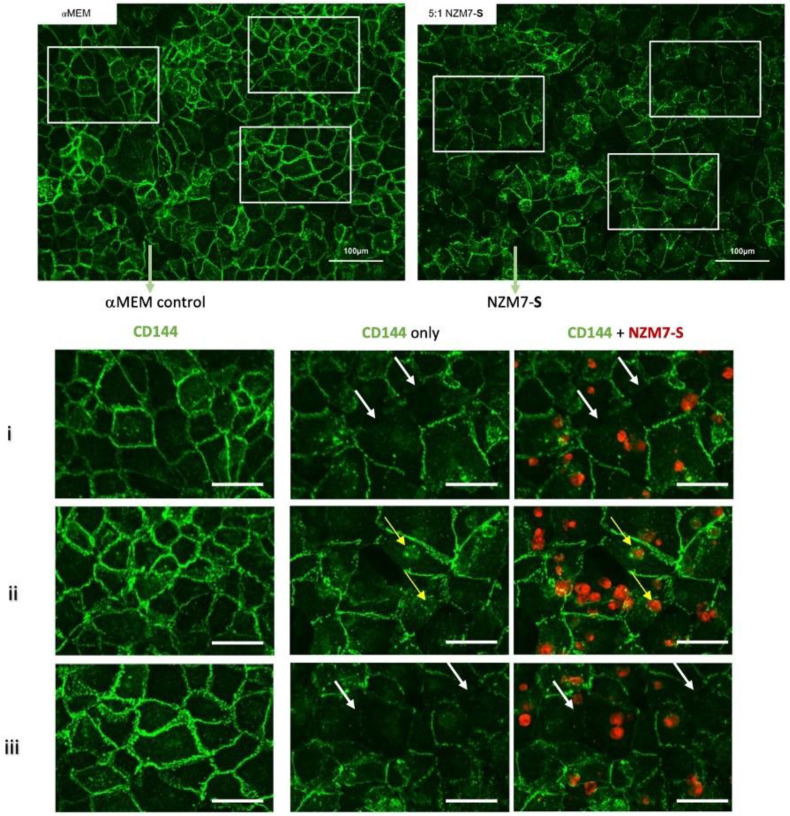
Expression of CD144 following the addition of 100,000 NZM7-S events to human cerebral microvascular endothelial cells. hCMVECs were seeded at 20,000 cells per well. After 48 h NZM7-S (red) was added in αMEM media and co-cultured for 4 h. Panels i–iii show expanded sections of the white boxes. Single left panel in i–iii shows αMEM control, and the two right panels show melanoma treated images. White arrows indicate lack of CD144 on cell borders. Yellow arrows indicate cytoplasmic expression of CD144 underneath NZM7-S. Images were acquired on the Operetta CLS imaging system. Full view images scale bar = 100 µm, cropped images scale bar = 50 µm. Images are representative of 2 independent experiments.

We next imaged the changes in endothelial junctional proteins, post melanoma addition. Figure 6 reveals the normal distribution of CD144 in healthy hCMVEC cells (Figure 6A) and distinct loss of CD144 localisation in the hCMVECs following addition of the NZM7-S population in an E:T ratio-dependent manner. The NZM7-S population was pre-labelled with CTR (red), and the figure shows the same images with and without the NZM7-S overlay to reveal a more global loss of CD144. These experiments were conducted in parallel with ECIS experiments, which show the barrier loss in a concordant manner. Figure 7 shows higher resolution images to reveal that the NZM7-S populations were present throughout the endothelial cells rather than positioned on the junctions. In addition, this highlights the widespread loss of CD144 (white arrows) and evidence of junctional disorganisation and co-localisation with NZM7-S, highlighted by the yellow arrows. We saw very little evidence of endothelial cell death, which implies that the NZM7-S population affects endothelial structural integrity rather than cytolysis. The scale bar in the small panels in Figure 7 is 50 µm, which gives the first glimpse of the large size of the melanoma-derived population inside the endothelial cells.

**Figure 8:**
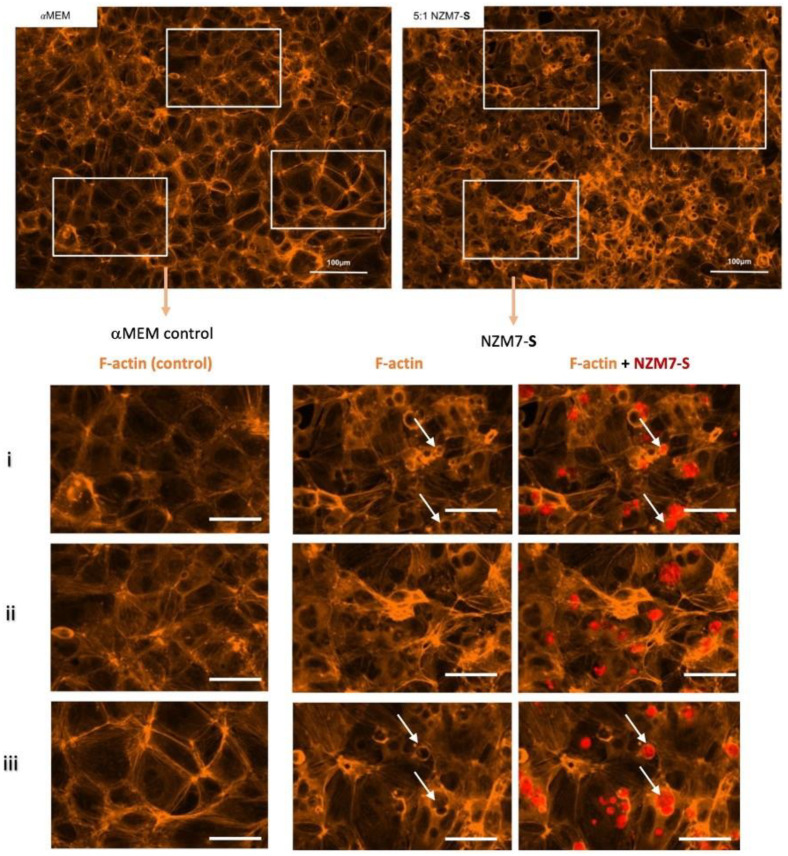
Expression of F-actin following the addition of 100,000 NZM7-S events to human cerebral microvascular endothelial cells. hCMVECs were seeded at 20,000 cells per well. After 48 h NZM7-S (red) was added in αMEM media and co-cultured for 4 h. Panels i–iii show expanded sections of the white boxes. Single left panel in i–iii shows αMEM control, and the two right panels show melanoma treated images. White arrows show ring-like F-actin structures. Full view images scale bar = 100 µm, cropped images scale bar = 50 µm. Images are representative of 2 independent experiments.

In these brain endothelial cells, typically F-actin is arranged in a highly organised fashion in close proximity to the cell membrane, close to the paracellular junctional boundary. However, following addition of the NZM7-S population, we saw a dramatic alteration in F-actin localisation. Some NZM7-S particles were clearly enveloped by actin filaments, and there was substantial re-arrangement of the F-actin from the paracellular membrane (Figure 8). This dramatic re-arrangement and removal of F-actin from supporting the cytoplasmic structure is consistent with the dramatic loss of endothelial barrier function measured by ECIS.

### 2.4. Live-Cell Imaging Shows NZM7-S Material Disrupting the Brain Endothelial Cells in a Transcellular Manner

**Figure 9:**
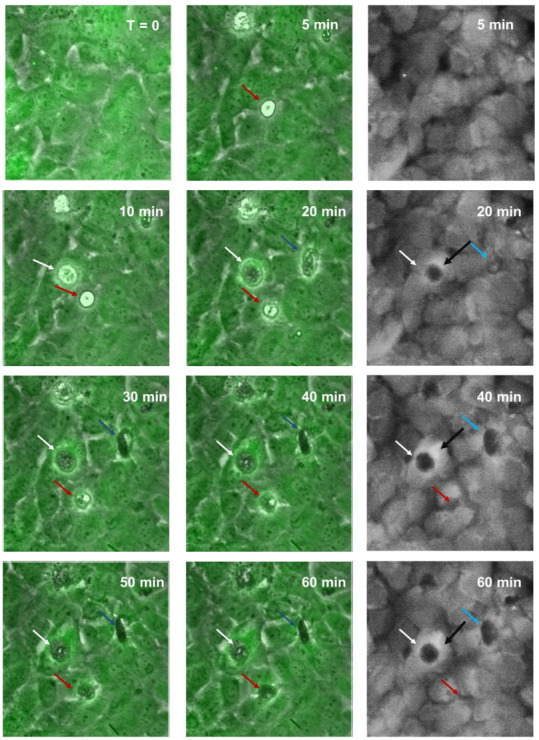
Time-lapse series illustrating melanoma integrating into brain endothelial layer. Images show endothelial cells stained with CMFDA-green, merged with phase only to visualise unstained spherical melanoma population (NZM7-S). Coloured arrows indicate NZM7-S that are taken up by endothelial cells. Monochrome (grey) images show green channel only to illustrate holes or pores forming within the endothelial cell body. Images shown are representative of 2 independent experiments.

The loss of paracellular and basolateral barrier at the same time and supporting imaging data suggested that the NZM7-S take a different route of migration at the endothelium, perhaps going through the endothelial body, to then disrupt both paracellular and basolateral components of the barrier (transcellular migration). We conducted live-cell imaging to visualise NZM7-S interacting with brain endothelial cells in real-time. Figure 9 shows the continuous endothelial monolayer attached at the cell to cell junctions, revealed by the green cytoplasmic stain (CMFDA). Monochrome images show the green filter only, to emphasise changes in endothelial cell monolayer upon melanoma addition (Figure 9).

Together, these images reveal that unstained NZM7-S (tracked by arrows) formed pores within the endothelial cell body within 20 min of addition (white arrows). As with the ECIS data, this was considerably faster than that seen with the adherent cells previously (first few hours of addition [55]). This increased over time for most (blue arrows) but not all NZM7-S (red arrows). The black arrows on the monochrome images point to the bright rings around the edge of the pores, showing that the endothelial cell shape had changed at the locus of invasion and was raised above the rest of the cell body. This is consistent with the change in the ring-like structure of F-actin observed previously. Supplemental Video S1 shows a real-time example of this to demonstrate that the NZM7-S were rapidly incorporated by the brain endothelial cells soon after addition, resulting in endothelial monolayer disruption. There was no morphological evidence of endothelial cell rupture and death. However, NZM7-S internalisation led to a drastic loss of the endothelial monolayer structure evident by both live-cell imaging and ECIS.

### 2.5. Confocal Microscopy Reveals Intricate Details about NZM7-S Interaction with the Brain Endothelial Cells

Next, we conducted high resolution confocal microscopy using a Zeiss LSM 800 Airyscan confocal. This imaging was incredibly informative, as it revealed detail not evident previously. Specifically, it (i) confirmed the widespread change in CD144 localisation, especially at high E:T ratios; (ii) provided definitive evidence that the NZM7-S material was inside the endothelial cells; (iii) provided a better appreciation of the range of sizes of the NZM7-S population inside the hCMVEC cells; (iv) revealed the close proximity of NZM7-S with the hCMVEC nuclei; (v) confirmed that endothelial cell death was not occurring; and (vi) revealed that some larger NZM7-S contained small amounts of DNA.

**Figure 10:**
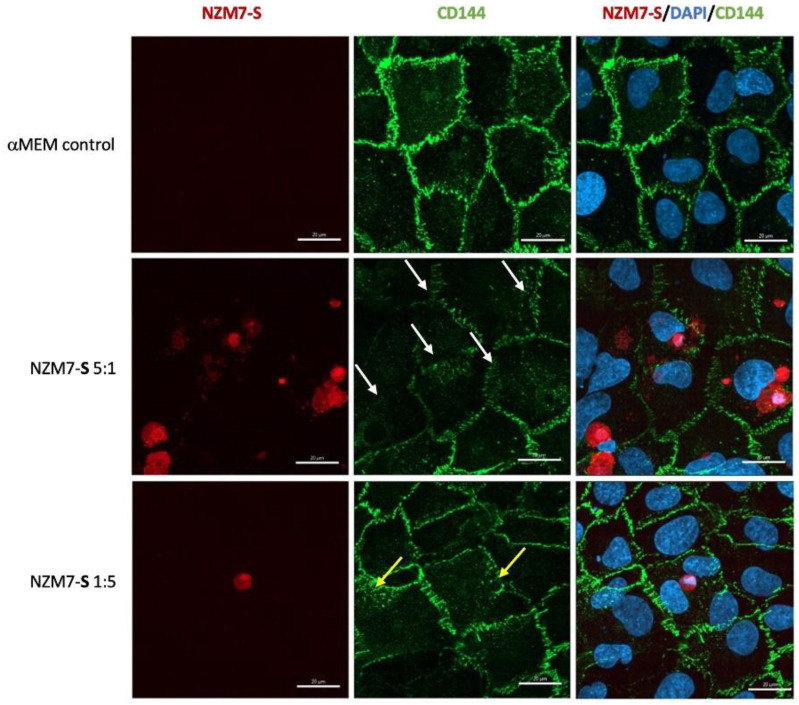
Representative images displaying changes in CD144 expression in human cerebral microvascular endothelial cells after NZM7-S addition detected by confocal microscopy. hCMVECs were seeded at 20,000 cells per well, and NZM7-S were live stained with CytoTrack Red (CTR) and applied to the apical face of a confluent endothelial monolayer. Cells were co-cultured for 4 h, washed and then fixed. Two E:T ratios of 5:1 and 1:5 are shown, labelled on the left. Media control was αMEM. Junctions were visualised with anti-CD144 (green), and nuclei were visualised using Hoechst. White arrows highlight stretched junctional borders. Yellow arrows highlight punctate CD144 staining within the endothelial cell body. Images were acquired with the Zeiss LSM 800 Airyscan confocal microscope. Scale bars = 20 µm.

**Figure 11:**
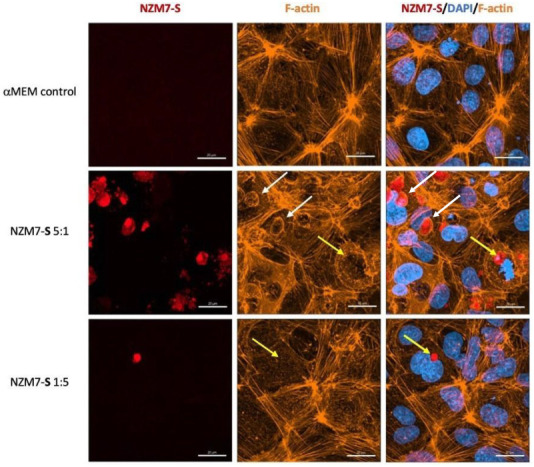
Representative images displaying changes in F-actin expression in human cerebral microvascular endothelial cells after NZM7-S addition detected by confocal microscopy. hCMVECs were seeded at 20,000 cells per well, and NZM7-S were live stained with CytoTrack Red (CTR) and applied to the apical face of a confluent endothelial monolayer. Cells were co-cultured for 4 h, washed and then fixed. F-actin was stained with Phalloidin, and nuclei were visualised using Hoechst. Two E:T ratios of 5:1 and 1:5 are shown, labelled on the left. Media control was αMEM. White arrows show ring-like F-actin structures surrounding NZM7-S. Yellow arrows show NZM7-S lacking F-actin ring structures. Images were acquired with the Zeiss LSM 800 Airyscan confocal microscope, scale bar = 20 µm.

Figure 10 confirms the substantial loss of CD144 and changes to the junctional organisation, where localisation of CD144 appears to be considerably more speckled and punctate (see white and yellow arrows, respectively). This is consistent with loss of junctional integrity. Figure 11 highlights the highly disorganised F-actin structures following the addition of NZM7-S.

**Figure 12:**
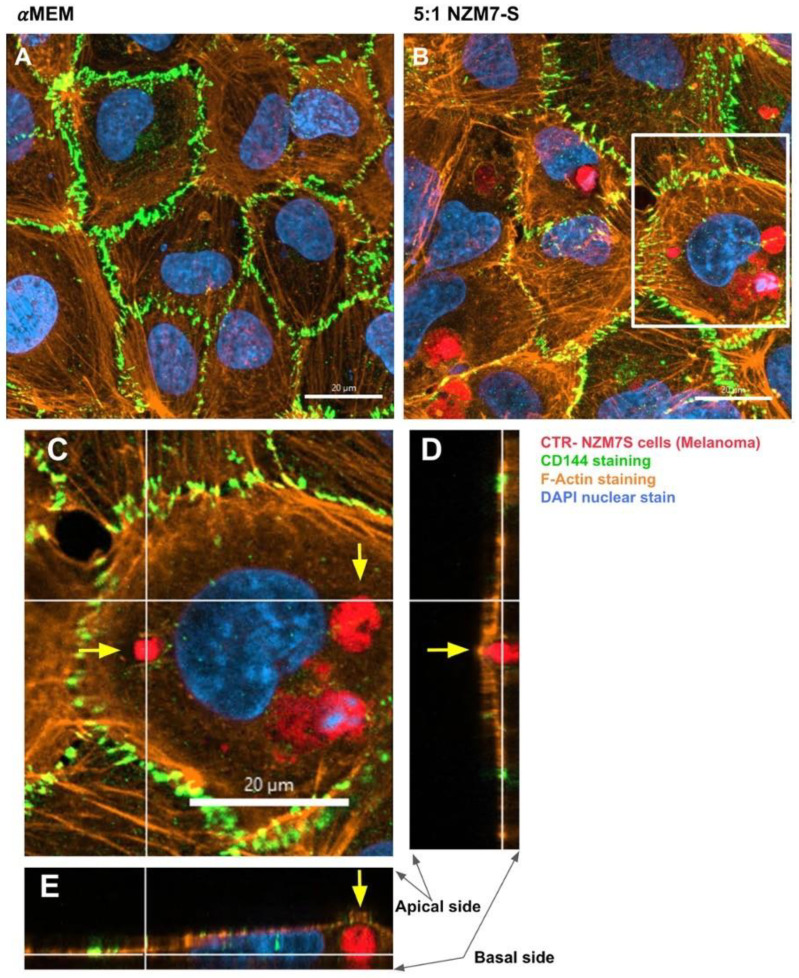
Confocal microscopy Z-stack imaging to localise the position of NZM7-S within cerebral endothelial cells. hCMVECs were seeded at 20,000 cells per well. NZM7-S were live stained with CytoTrack Red (CTR) and applied to the apical face of a confluent endothelial monolayer. Cells were co-cultured for 4 h, washed and then fixed. Junctions were stained with anti-CD144 (green), actin stained with phalloidin (orange) and nuclei stained with Hoechst (blue). Samples were analysed by confocal laser scanning microscopy. (**A**) Untreated hCMVECs. (**B**) NZM7-S added at a 5:1 E:T ratio; the white box is expanded in (**C**). (**C**) The xy-stack at the level of the white lines on (**D**,**E**). (**D**,**E**) The z-stacks along the vertical and horizontal line, respectively, on (**C**). Yellow arrows show NZM7-S located within the cerebral endothelial cell body underneath endothelial actin. Images acquired using a Zeiss LSM 800 Airyscan confocal microscope.

**Figure 13:**
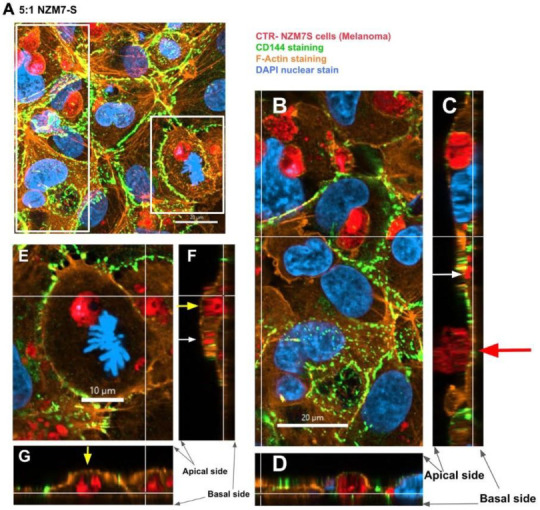
Confocal microscopy Z-stack image of different sized NZM7-S and their localisation. hCMVECs were seeded at 20,000 cells per well. NZM7-S were live stained with CytoTrack Red (CTR) and applied to the apical face of a confluent endothelial monolayer. Samples were analysed by confocal laser scanning microscopy. (**A**) The xy-stack of NZM7-S added at an E:T ratio of 5:1. Boxed regions are magnified to the right of, (long, left box) and below (short, right box) image A. (**B**) The xy-slice at the level of the basal white lines on (**C**,**D**). (**C**,**D**) The z-stacks along the vertical and horizontal line, respectively, on B. (**E**–**G**) The same xyz stack configuration. Yellow arrows show large NZM7-S inside the endothelial cell body. White arrows show small NZM7-S encapsulated by actin filaments of the hCMVEC. Red arrow shows NZM7-S cell positioned on the apical surface of the endothelial cell body. Images acquired using a Zeiss LSM 800 Airyscan confocal microscope.

**Figure 14:**
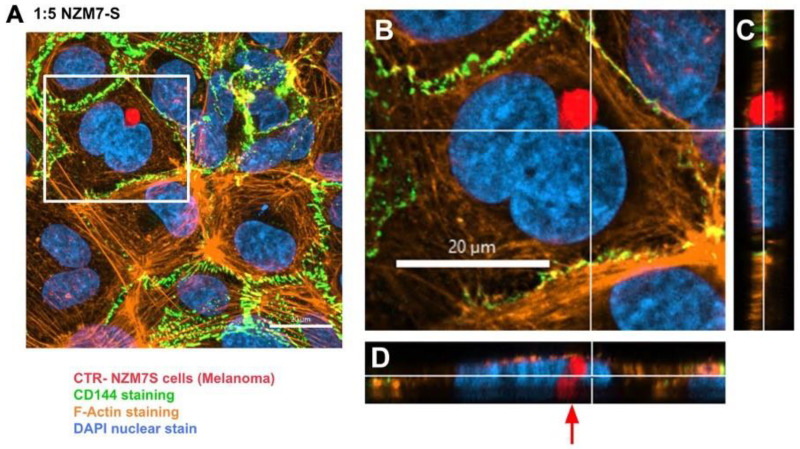
Confocal microscopy Z-stack image of NZM7-S position localised to the hCMVEC nucleus. hCMVECs were seeded at 20,000 cells per well. NZM7-S were live stained with CytoTrack Red (CTR) and applied to the apical face of a confluent endothelial monolayer. Samples were analysed by confocal laser scanning microscopy. (**A**) The xy-stack of NZM7-S added at an E:T ratio of 1:5. (**B**) The xy-stack at the level of the white lines on (**C**,**D**). (**C**,**D**) The z-stacks along the vertical and horizontal line, respectively, on (**B**). (**A**) The NZM7-S positioned within the endothelial cell body next to the hCMVEC nucleus. Red arrow indicates interesting finding of NZM7-S close to the nucleus. Images acquired using a Zeiss LSM 800 Airyscan confocal microscope.

In addition, several large species of NZM7-S that were enveloped by F-actin are highlighted with white arrows. Other species of NZM7-S were not enveloped by F-actin but are juxtaposed to the endothelial nucleus (yellow arrows, Figure 11). Such an observation raises concerning speculation as to the functional association of the NZM7-S species with the endothelial nucleus. Regardless of such, it is clear that the NZM7-S material was having a devastating impact on the structural integrity of the endothelial cells. Figure 12 reveals in intricate detail the positioning of multiple NZM7-S inside the same endothelial cell, where the NZM7-S is immediately below the hCMVEC cell membrane and appears to be pushing it upwards. This may imply the NZM7-S sub-population is not easily compressible, which is consistent with them being very dense. Notably, dense packaging is also a characteristic of extracellular vesicular materials, as detailed in the introduction. It also confirms the presence of what appears to be fragmented DNA in some of the large NZM7-S species, which is also characteristic of vesicular cargo. In Supplemental Figure S5, Hoechst-based staining of the NZM7-S particles shows that NZM7-S took up both cell-permeable and cell-impermeant nuclear dyes readily, further supporting their ability to carry nuclear content. Some NZM7-S were positive for “live-cell” nuclear stains (blue arrows), but several were positive for both live and dead nuclear stains as indicated by the teal arrows. This was surprising and suggested that NZM7-S had a nucleus (stained blue), and some of these cells had a compromised membrane (green arrow). Supplemental Figure S6 shows that all NZM7-S material greater than 2.5 µm had detectable nuclear material, and fluorescence intensity of the nuclear dye increased with the NZM7-S cell size. The results seem reasonable if we note that the concoction of materials in the NZM7-S was likely a mixture of apoptotic melanoma cells, apoptotic bodies (vesicular ApoBDs) and other large extracellular vesicles such as large oncosomes. The NZM7-S highlighted in Figure 13 is particularly intriguing, as it was attached to the outside of the endothelial cells (Figure 13B–D). Additionally, Figure 13E–G show a large NZM7-S juxtaposed to the nucleus of the endothelial cell, where the nucleus is dividing or fragmenting. Another NZM7-S is shown in Figure 14, which appears to be intimately associated with the nucleus. The confocal images provide clear evidence that the NZM7-S population was not intact melanoma cells. None of the vesicular-bodies had intact nuclei, but some had small amounts of DNA that appeared condensed and fragmented. The confocal Z-stacks also revealed that the NZM7-S were often spherical and intensely stained with CTR. This implies that the NZM7-S are enzymatically competent and enveloped by cell membrane, much like a very large vesicle. Clearly, there was a range of sizes, with many around 5–10 µm in diameter, and some were much smaller and some larger. Such a size and morphology is consistent with apoptotic bodies and large-oncosomes, which are both classified as cellular vesicles [46].

### 2.6. ECIS Measurements Show That CTR++ NZM7-S Material Decrease the Brain Endothelial Barrier Resistance

Previously, we presented in Figure 1 that the flow cytometry scatter profile of the collected NZM7-S material showed the presence of minimal NZM7A as well. As we have never seen such a drastic effect on the basolateral compartment (Alpha) with the NZM7A, it was prudent to separate the two cell types to attribute the disruption to the NZM7-S material alone.

**Figure 15:**
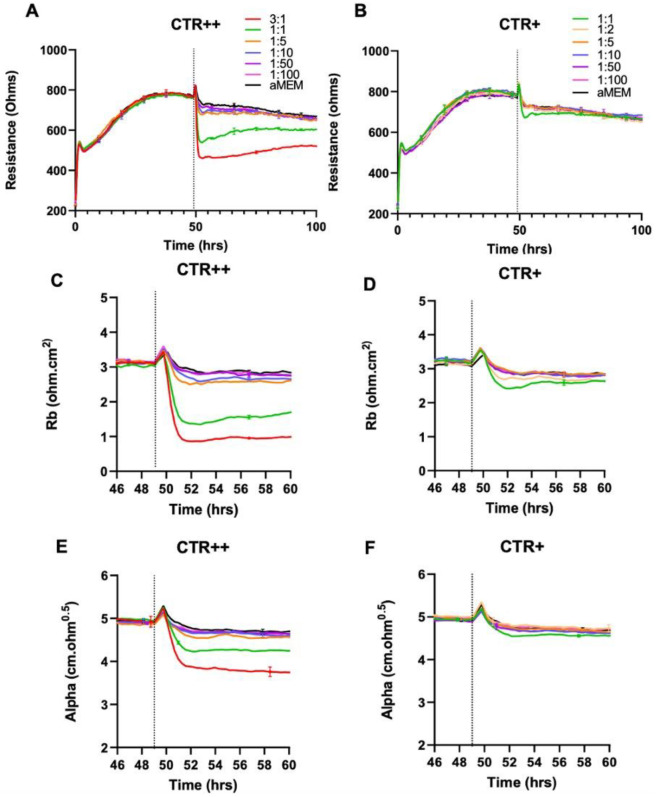
Effect of FACS-isolated CTR++ and CTR+ NZM7-S material on brain endothelial barrier resistance. (**A**) Unmodelled resistance (at 4000 Hz) of isolated CTR++ population of NZM7-S material, (**B**) Unmodelled resistance (at 4000 Hz) of isolated CTR+ population of NZM7-S material. (**C**,**D**) Changes in the paracellular barrier (Rb) of CTR++ and CTR+, respectively. (**E**,**F**) Changes in the basolateral barrier (Alpha) of CTR++ and CTR+, respectively. Cells were added at different E:T ratios, where 1:1 shows 1 NZM7-S event as counted with the Accuri C6 flow cytometer to 1 endothelial cell. NZM7-S material was added at 49 h. Data shown as mean ± SD (*n* = 3 wells) from 1 experiment, which is representative of 2 independent experiments.

To accomplish this, we utilised the CTR staining intensities of the NZM7-S material to sort the populations. Supplemental Figure S7 shows that FACS sorted data removed most of the adherent population with less than 0.3% remaining. Earlier we had noted that the NZM7-S population had a bi-model distribution for labelling with CTR. Therefore, we chose to further FACS sort the NZM7-S population on the basis of CTR intensity. The flow cytometry data are shown in Supplemental Figure S8, and two populations were collected. Both were positive for the CTR stain, with the CTR+ population considerably less intense than the CTR++ population. Note that the sorting gates on the Aria II were set up to also exclude the living melanoma cells from the sorted populations. Immediately after sorting, the respective samples were counted and added at a range of E:T ratios to identify which sample had the greater effect on endothelial barrier integrity. In order to do this, hCMVECs were set up on the ECIS system with precision timing that their barrier formation would coincide with the production of the sorted NZM7-S population. Figure 15 reveals that the intensely labelled CTR++ NZM7-S population was considerably more potent than the CTR+ samples.

Thus, the population that had the greater enzymatic activity and ability to retain the CTR stain (CTR++) caused a much greater degree of disruption to the endothelial cells as measured by ECIS (Figure 15). Note that the 1:1 ratio represents the same number of particles added to the hCMVECs. We would estimate, based on the relative E:T ratios, that the disruption caused by the CTR++ material was five times greater than the CTR+ population. This therefore provides a first step of enrichment of the NZM7-S bioactivity, which will be relevant for full characterisation of this melanoma-derived population.

## 3. Discussion

In this research, we demonstrate the potent barrier-disrupting activity of melanoma-derived material, which is distinct from intact living melanoma cells. We demonstrate that the material closely resembles a range of large vesicles that (i) are surrounded by cell membrane, (ii) are enzymatically active, and (iii) exist in a range of sizes with some smaller than 1 µm and some larger than 10 µm, with a proportion of the larger species containing DNA. For simplicity at this juncture, we have termed these species melanoma-derived vesicular-bodies. We have generated this terminology for our paper, as the materials were not collected with the current classical EV collection protocol. We suggest the importance of these large non-adherent cell-like material, collected after the 500× *g* spin, which is typically discarded during EV isolation to remove large cell debris. Crucially, we reveal that this concoction of large vesicular-bodies affects the barrier integrity of brain endothelial cells within an hour of exposure, significantly faster and more drastically than their adherent counterpart (Supplemental Figure S4). This occurs in a manner that involves global and extensive loss of CD144, substantial re-arrangement of F-actin and simultaneous loss of basolateral adhesion and paracellular junctional integrity, as shown in the modelled ECIS data. We show that the mechanism of disruption appears to be mediated by uptake of the vesicular-bodies by the endothelial cells, which is completely distinct to that mediated by the melanoma cells proper [55]. Confocal imaging demonstrates that the vesicular-bodies do not kill the brain endothelial cells, but rather they subvert the F-actin cytoskeletal structure and cause widespread changes of CD144 from the junctions. Intriguingly, the melanoma-derived large vesicular-bodies are often located juxtaposed to the nucleus, which could be suggestive of some form of influence of the vesicle’s cargo with the nucleus. While this finding raises speculations on how the NZM7-S interacts with the endothelial cell, the current literature on cancer cell transcellular transmigration lacks any supporting evidence of this. In the case of leukocytes, however, an early study by Ushiki [68] on the migration of leukocytes into the thymus using a rat model provided evidence of transcellular transmigration, noting the route of diapedesis occurred centrally through endothelial cells in peri-nuclear locations.

More recent evidence shows that leukocytes adapt a stochastic mechanism to extravasate transcellularly, to allow travel through locations of relatively low endothelial resistance, via a podosome-mediated mechanism [69]. The NZM7-S proximity to the nucleus may be a consequence of the relatively large size of the nucleus. Carman and colleagues also noted the appearance of podoprints and invaginations over nuclei and suggested leukocytes decipher the route of transmigration through palpation and trial and error. This could suggest a potential mechanism of transmigration by the melanoma subpopulation, as tumour cells are known to use invadopodia to degrade local microenvironments and overcome cellular barriers that prevent cells from spreading [70]. Invadopodia are however very complex cellular structures. Their potential involvement in NZM7-S transmigration is less likely due to the smaller phenotype of the suspension material. Furthermore, Carman et al. (2007) only visualised podoprints over nuclei, whereas we observed the entire material engulfed, suggesting a different mechanism. Our data demonstrate that the melanoma suspension population is not comprised of intact cells, but rather it resembles very large vesicular-bodies consistent with those reported for large oncosomes and large apoptotic bodies [41,71,72], which are each classified as being cell-derived vesicles [36].

The decision was taken early to pursue the **functional importance** of the NZM7-S material on the integrity of the brain endothelial cells. This was because we hypothesised that the NZM7-S were melanoma cells transforming into more metastatic cells. This was an intriguing consideration, as a more metastatic phenotype would be consistent with the cells becoming less adherent and more invasive. However, our initial flow cytometry analysis indicated that the NZM7-S population was not alive, as its forward and side scatter profiles were considerably different than those of the living melanoma cells. In addition, its side scatter profile was much larger, indicating a considerably greater density and complexity, implying substantial changes in the cell morphology. The fact we could easily stain the NZM7-S population with CTR live-cell stain indicated an abundance of esterase activity and an intact cell membrane.

ECIS technology represents the most sophisticated technology for measuring real-time changes in endothelial barrier strength [67]. Moreover, the sophisticated mathematical modelling of ECIS data enables assessment of barrier-related changes at the paracellular compartment independently from changes occurring with the basolateral adhesion of the cells [55,62,67]. This is eloquently demonstrated with the living, intact melanoma cells that affect the paracellular barrier (Rb) several hours prior to their effect on the basolateral adhesion (Alpha) of the endothelial cells [55]. In contrast, the NZM7-S material affected the paracellular barrier and the basolateral adhesion **simultaneously** and considerably faster than intact melanoma cells. The obvious explanation for such a dramatic effect, was death of the endothelial cells. Confocal imaging revealed that this was not the case, but rather that the NZM7-S vesicles were internalised within the endothelial cells and caused substantive rearrangement of F-actin. Notably the F-actin was removed from its normal circumferential location around the periphery of the endothelial cells, where it is thought to provide structural integrity [73,74]. An interesting observation was the appearance of cup-like actin structures present around many of the NZM7-S within the endothelial cell body. F-actin rings have a documented role in leukocyte diapedesis, rather than leukocyte adhesion [75,76]. The assembly of these structures is followed by the continued formation of either a gap (paracellularly) or a pore (transcellularly) to allow for leukocyte diapedesis through the tight cytoskeletal structure of endothelial cells [77]. Dense F-actin ring-like structures are mechanistically involved in maintaining endothelial cell integrity during leukocyte transendothelial migration [74]. However, here the results show a lack of maintained cellular integrity, as evident through the highly destructive effects of the NZM7-S material on the endothelial barrier strength. A recent study by Godinho-Pereira, Garcia [78] demonstrated enhanced transmigration of breast cancer cells through the formation of actin stress fibres, inducing endothelial cell permeability via cytoskeletal rearrangement.

In healthy endothelial cells, the F-actin fibres are localised to the circumference of the cell body and are densely positioned at multicellular junctions to maintain cellular integrity and barrier integrity [76]. The induction of actin stress fibres is essential to facilitate cellular movement [79]. Here, the high-resolution imaging of F-actin shows the complete disassembly of circumferential F-actin at high E:T ratios of NZM7-S treatment, the loss of multicellular junctional F-actin and the increase of disorganised actin stress fibres across the cells. In conjunction, we observed dramatic and widespread loss of CD144 from the paracellular junctional space, even in regions where there were no obvious NZM7-S vesicles located. This included junctions where CD144 was present in diffuse puncta and other junctions where CD144 was barely detectible. This change in CD144 is a critical observation and is consistent with the loss of barrier integrity measured by ECIS. The actual molecular steps resulting in this are not yet known, but it is logical to associate the changes in F-actin with the loss of paracellular junctional regulation and localisation of CD144. It is also possible that the NZM7-S affect the endothelial cell integrity by delocalising CD144, which has been reported to modulate endothelial cell barrier dysfunction via internalisation [80]. Previous literature has suggested that activation and expression of Rab5 in endothelial cells in response to lipopolysaccharide (LPS) promotes CD144 translocation into intracellular compartments [81]. CD144 internalisation has also been shown under hypoxic conditions in human brain endothelial cells in vitro, resulting in increased permeability and damage to the barrier through a RhoA/ROCK2-mediated pathway [82]. Therefore, it is possible that the NZM7-S are acting on the cerebral endothelial cells, resulting in loss of junctional CD144 but also on the delocalisation and internalisation of this protein through yet unknown mechanisms.

The confocal imaging in this study has powerfully demonstrated the impact of the melanoma-derived vesicular-bodies on the integrity of the endothelial cells and has revealed key information pertaining to the nature of these materials. The original flow cytometry data show material with a wide range of sizes and complexities. This is also evident in the confocal images, where vesicles with a wide range of sizes are evident, where some of the larger species contain DNA. This is evidence of considerable heterogeneity in the species of vesicular-bodies present. We hypothesise that the material is most likely a combination of apoptotic melanoma cells, apoptotic cell-derived vesicles (ApoBDs) and other large extracellular vesicles such as large oncosomes released by the melanoma cells. Large oncosomes have only been described in a small subset of cancers, with the first discovery of these entities documented through a study by Di Vizio, Kim [83] on late-stage prostate cancer. The atypically large sizes of large oncosomes are the result of membrane blebbing and are associated only with advanced disease [43]. These highly enzymatically active vesicles have been shown in prostate cancer to be enriched in growth factors (i.e., TGF-β), cathepsin proteases and proteins related to cell adhesion [41]. Some of the melanoma-derived vesicular-bodies observed are consistent with the descriptions of large oncosomes. However, definitive methodologies for identifying, isolating, and purifying large oncosomes is a developing area, and there is no literature detailing their presence in melanoma as of yet. The literature suggests that each of these large vesicle species is derived from distinct cellular processes [28,71] and they are considerably heterogeneous in terms of molecular profiles [41,42] and functional relevance [83,84,85]. Their characterisation and assessment are significant processes, especially where there is some overlap in molecular expression profiles, or where their phenotypes are poorly understood.

Cancer cell-derived extracellular vesicles, apoptotic circulating tumour cells (CTC) and CTC debris have been documented in blood samples of patients with a variety of late-stage malignancies, including but not limited to breast, colon, prostate and pancreatic cancer [50,86,87,88]. The possibility that cancers deliberately release vesicles or utilise apoptotic cells in the circulation to prime sites for invasion is beginning to be identified as a reality in aggressive tumour cell progression. While melanoma exosomes and CTCs have been identified in late stage malignancy [31,89,90,91], the potential role of CTC debris in brain metastasis has yet to be elucidated.

## 4. Materials and Methods

### 4.1. Culture of Human Cerebral Microvascular Endothelial Cells (hCMVECs)

Human brain endothelial cells (hCMVECs) are an immortalised cell line that was purchased from Applied Biological Materials Inc. (ABM, Cat:T0259, Richmond, BC, Canada). The cells were cultured in 75 cm^2^ Nunc cell culture flasks and supplied with M199 growth media containing 10% FBS, 1 μg/mL hydrocortisone, 3 ng/mL hFGF, 1 ng/mL hEGF, 10 μg/mL heparin, 2 nM GlutaMAX and 80 μM dibutyryl-cAMP (suppliers detailed previously in [55]). Prior to cell plating, all flasks used for cell maintenance and experiments were coated with 1 μg/cm^2^ collagen I in 0.02 mM glacial acetic acid for an hour and then washed 3 times with sterile MilliQ water. Collagen coated plates were stored at 4 °C and used within 1 month of preparation.

### 4.2. Culture of New Zealand Melanoma (NZM) Cells

Each of the different melanoma cell cultures were originally developed by Professor Bruce Baguley and his team in Auckland Cancer Society Research Centre and used in collaboration with Dr. Graeme Finlay. Three melanoma cell lines, which were all originally from melanoma patients, were NZM7, NZM48 and NZM74 (Research Resource ID: CVCL_D843, CCVL_S423 and CVCL_0D38, respectively). The melanoma cell lines were cultured in both Falcon T75 and T25 flasks with Minimum Essential Media α (αMEM) containing 5% FBS, 5 μg/mL insulin, 5 μg/mL transferrin and 5 ng/mL sodium selenite (ITS), in normoxic conditions (suppliers detailed previously in [55]).

### 4.3. Cell Harvest and Passaging

Both cell types (hCMVEC, NZM) were harvested after reaching 90–100% confluency in their respective culture flasks. Cells were treated with 1× TrypLE Express (Gibco, Thermo Fisher Scientific, Cat:12605010, Waltham, MA, USA) for 3–5 min and were then centrifuged at 300× *g* for 5 min to collect the cell pellet. It is important to preserve the cell-surface phenotype including adhesion molecules on melanoma cells that may aid melanoma cell adhesion. Therefore, TrypLE was used throughout all cell cultures to maintain their cell surface phenotype. The NZM cultures were not used past passage 35 in order to minimise the potential for increasing mutational load of the melanoma cells.

### 4.4. Collection of NZM7 Suspension Material

NZM Suspension material (NZM7-S) was collected from the NZM7 cell line. NZM7 cells were seeded at a density of 1,000,000 cells in a T75 flask. Suspension material was collected from the cells on day 7–8 (~100% confluency), whilst minimising collection of the adherent cells. The collected media suspension was centrifuged for 10 min at 500× *g*, and the supernatant was removed. Note that this method of collection produces a pellet containing large cellular material and is distinct from the classical extracellular-vesicle (EV) collection protocol. The resultant pellet was then harvested and is referred to as the NZM7-**S** material. Their parent cell line was called NZM7**A** for **adherent** cells. For melanoma lines NZM48 and NZM74, adherent cells were grown for as long as required to detect and collect their suspension material, provided that the adherent population was still viable. NZM74 did not produce sufficient material; however, NZM48 did after ~10 days. Hence, additional NZM lines were added for supplemental data, named NZM41 (CVCL_S426) and NZM98 (CVCL_0D58).

### 4.5. Bright Field Imaging of Cells

Images of the NZM7-S and NZM7A cells were taken using the EVOS FL auto imaging system (Invitrogen, Cat:AMAFD1000, Waltham, MA, USA). Cells were imaged with phase microscopy in their culture flask and imaged at 10× and 20× magnification.

### 4.6. Flow Cytometry

Flow cytometry using an Accuri C6 flow cytometer (BD Biosciences, Franklin Lakes, NJ, USA) was used for initial basic characterisation of the NZM cells (NZM7A) and cell material (NZM7-S). NZM7A cells were harvested and stained with 500 nM CellTracker™ Green 5-chloromethylfluorescein diacetate (CMFDA) (Invitrogen, Cat:C7025) for 30 min in serum-free media. Note that 500 nM CMFDA was used, as higher concentrations were too bright for flow cytometry, resulting in rightward-shifted cells located off the 7-log scale (Supplemental Figure S1). After labelling, the NZM7 cells were washed twice with 1 mL FACS buffer (1% FBS + PBS at 4 °C), centrifuged after each wash at 300× *g* for 5 min to secure the stained cell pellet, leaving 100 μL of FACS buffer for re-suspension and running through the Accuri C6 flow cytometer. NZM7-S were harvested as above, where the NZM7-S were stained with 250 nM CytoTrack™ Red (CTR) (Bio-Rad, Hercules, CA, USA, Cat:135-1205) for 30 min in serum-free media. After this time, 1 mL of FACS buffer was added to the sample to quench any free dye, and the samples were centrifuged at 500× *g* for 10 min to secure the stained pellet, leaving 100 μL of FACS buffer for resuspension and running through the Accuri C6 flow cytometer. Unstained populations were also prepared omitting the respective staining steps.

### 4.7. Conventional Analysis and Gating

Sample analysis was carried out using BD Accuri C6 software v.1.0.264.21. Information regarding the size and density of the NZM7-S melanoma sub-population was gathered to assess the nature of the material in comparison to melanoma cells proper. This was displayed as forward scatter (FSC; cell size) and side scatter (SSC; cell density), as analysed by the Accuri C6 software v.1.0.264.21 and displayed digitally as an “FSC vs. SSC” scatterplot. The fluorescence measured for each melanoma population was determined as a median fluorescent intensity (MFI) and plotted against the cell count on a logarithmic scale. CMFDA and CTR were used respectively as viability stains for the NZM7A and NZM7-S populations. The cell-tracker fluoresces after crossing the cell membrane and being enzymatically cleaved by esterase activity within the cell, indicating enzymatic activity and a biologically active function.

### 4.8. Electric Cell-Substrate Impedance Sensing (ECIS)

Electrical Cell-Substrate Impedance Sensing (ECIS) technology allows for real-time and label free assessment of a number of barrier-related parameters in response to changes in cellular behaviour. The impedance-based technology is able to apply a low alternating current (AC) through a confluent monolayer of cells, where the potential is then detected by ECIS technology and is used to calculate the impedance. The flow of electrons through the endothelial cell monolayer varies at different frequencies of AC and allows for the measurement of different properties of the barrier, including resistance and capacitance [62]. At low frequencies, varying between 2000 and 4000 Hz, the membrane resistance is high. High resistance results in most of the current being unable to flow through the cells and opting to take the path of least resistance, which is between the cells. At a frequency of 4000 Hz, the flow of current acts upon the intercellular space between the endothelial cells, which represents the cell–cell junctions. Therefore, at this frequency, any changes in resistance can provide information on the integrity of the endothelial barrier through the junctional spaces of the monolayer [67].

To assess the strength of the cerebral endothelial barrier, hCMVECs were grown on gold-plated 96-well ECIS plates (Applied Biophysics, Troy, NY, USA). As the barrier-forming endothelial cells attach and begin to form a monolayer, the resistance to current flow increases. The current flow becomes restricted following the formation of a confluent layer of cells and results in a plateau of the measured resistance once the barrier is formed. The plateau is what is then referred to as the **barrier resistance** and represents the integrity and strength of the formed brain endothelial barrier.

### 4.9. ECIS Set up

The ECIS Zθ system 96-well plates (item 96W20idf) were used, which were lined with interdigitating gold-plated electrodes. Prior to the experiment, the 96-well plates were coated with 10 nM cysteine to stabilise and maintain electrode capacitance as per manufacturing guidelines. After 15 min, the cysteine was removed and washed three times with sterile MilliQ water. Following the cysteine coat, the wells were then coated with 1 μg/cm^2^ of rat-tail collagen I in 0.02 M acetic acid as per hCMVECs cell culture protocol (Section 4.1).

The hCMVECs were seeded at a density of 20,000 cells per well in 100 μL of complete M199 growth media. Each seeded ECIS plate was then attached to the ECIS machine and incubated at 37 °C. The machine was programmed to record continuously at multi-frequency, allowing for the ECIS system to record resistance across low and high frequencies and model the recorded resistance into separate components.

### 4.10. ECIS Modelling

ECIS technology is able to mathematically model the overall resistance into two separate parameters of barrier integrity as a result of the system’s ability to apply AC at multiple frequencies. When determining overall resistance between a monolayer of endothelial cells, both the inter-endothelial (paracellular) and basolateral components comprise the total resistance value. The two components are then defined by the ECIS software v1.2.163.0 as resistance beta (Rb) and resistance alpha (Ra/Alpha). Rb is the paracellular component of the endothelial barrier that is created by the junctional molecules, including tight junctions of the neighbouring cells, while Alpha is the basolateral component created by the basolateral membrane of the cells attached to the electrode below (Figure 2).

### 4.11. Addition of Melanoma Cells to hCMVECs

The NZM7A cells were harvested carefully using TrypLE Express to maintain their cell surface phenotype. The NZM7A cells were then added to the hCMVECs on the ECIS biosensor at five different Effector:Target (E:T) ratios, namely, (1:1), (1:5), (1:10), (1:50) and (1:100), where (1:1) represents 1 melanoma cell per endothelial cell. For example, each well had 20,000 endothelial cells added; therefore, an E:T ratio of 1:1 = 20,000 melanoma cells added to 20,000 endothelial cells; an E:T ratio of 1:10 = 2000 melanoma cells added to 20,000 endothelial cells. All E:T ratios were added in 100 μL of complete αMEM media per well. The media control was comprised of 100 μL of only complete αMEM, also added to the hCMVECs. The use of different E:T ratios allowed for the understanding of the sensitivity of the system to assess the lowest effector number that still provides a measurable effect on the endothelial cells (detected by the ECIS biosensor).

### 4.12. Addition of Suspension Material to hCMVECs

The NZM7-S population was prepared in 100 μL of complete αMEM media and added at a range of E:T ratios. The effective E:T ratios varied between experiments due to the total yield of material. The suspension material was run through a flow cytometer and the number of events counted. This was used to work out the number of particles per µL, and the best dilutions were generated depending on the number of particles collected. Hence, there was variability in titrations added across assays. This proved to be more accurate than counting with a microscope or haemocytometer. Typically, the target ratios were as follows: E:T = (5:1), (2:1), (1:1), (1:5), (1:10), (1:50), (1:100), where **E** is the number of NZM7-S, and **T** is the number of endothelial cells. ECIS data were analysed using GraphPad Prism software and plotted as the mean (± SD) from multiple replicates (typically 3 wells).

### 4.13. Labelling of the NZM7 Cultures for Imaging

In imaging experiments, NZM7A cells and NZM7-S material were pre-labelled in order to facilitate tracking during image analysis. NZM7A cells were stained with 1 μM CellTracker™ Green 5-chloromethylfluorescein diacetate (CMFDA) (Invitrogen, Cat:C7025) for 30 min in serum-free media. After this time, the cells were centrifuged for 5 min at 300× *g*, and the supernatant was discarded. The resultant CMFDA labelled cells were reconstituted in media and maintained at 37 °C until required for co-culture with the hCMVECs.

NZM7-S were stained with 1 μM CytoTrack™ Red (CTR) (Bio-Rad, Cat:135-1205) for 30 min in serum-free media. After this time, the NZM7-S material was centrifuged for 10 min at 500× *g*, and the supernatant was discarded. The CTR labelled material was used immediately in flow cytometry experiments or added to hCMVECs as detailed below or in the respective figure legend. Note that the concentrations of CMFDA and CTR used for imaging were slightly higher than that used for the initial flow cytometry analysis, as 1 µM labelled material was too bright for the flow analysis, and 1 µM was better for image tracking, especially for confocal.

### 4.14. Immunocytochemistry

hCMVECs were seeded in µ-Plate 96-well, black plates (Cat: 89626, Ibidi, Bayern, Germany) coated with 1 µg/cm^2^ of rat-tail collagen and 0.02 M acetic acid. Cells were seeded at a density of 20,000 cells per well and grown for 48 h for the formation of their barrier as detected with ECIS. NZMA and NZM7-S were stained with their respective cell-trackers, as detailed previously (at 1 µM), and then added to the hCMVECs for designated periods, which are indicated in the respective figure legends. The cells were fixed with 4% paraformaldehyde. Blocking was performed using 1% bovine serum albumin (BSA) for 45 min, followed by three washes with 0.1% Triton X-100 in phosphate-buffered saline (PBST).

The endothelial cells were stained for detection of VE-cadherin (mouse anti-human VE-cadherin, dilution 1:200, SantaCruz, Dallas, TX, USA, Cat:sc-9989). The antibodies were reconstituted in 1% BSA and incubated with the fixed co-culture for 4 h on a rocker. Post incubation, the primary antibody was washed 3 times for 10 min on a rocker with PBST. The goat anti-mouse secondary antibody conjugated to Alexa Flour 488 (Invitrogen, Cat:A-11001) was added along with an F-actin probe (rhodamine-phalloidin; dilution 1:50, ThermoFisher, Waltham, MA, USA, Cat:R37112) and Hoechst (Invitrogen, Cat:62249, 1:10,000 dilution) for 2 h. Following the final incubation, the samples were washed 3 times for 10 min on a rocker with PBST.

### 4.15. Live Cell Imaging

Live-cell imaging was conducted on the Nikon BioStation (Nikon Instruments Inc., Tokyo, Japan). Brain endothelial cells (400,000) were seeded in a 35 mm Petri dish (Nunc 153006, Roskilde, Denmark) in 1.5 mL of M199 growth medium. Cells were cultured as per growth conditions described above for 48 h to achieve confluency. In order to visualise the focal plane of the endothelial cells in real-time, the brain endothelial cells were stained with 1 µM CellTracker™ Green CMFDA (Thermo Fisher Scientific, Waltham, MA, USA) and imaged for 2 h, after which 40,000 NZM7-S cells (E:T of 1:10) were added in 500 µL of complete αMEM and imaged for a further 5 h. The software was programmed to acquire images (phase and GFP channel) every 5 min across multiple sites throughout the entire time-lapse experiment.

### 4.16. Operetta CLS Imaging System

Imaging was initially conducted using the Perkin Elmer Operetta High-Content Imaging System (PerkinElmer, Waltham, MA, USA), which is designed for high content imaging. The Operetta is equipped with a 20×/0.75 NA lens. The fluorophores used were specified during the imaging setup, allowing the instrument to select the most appropriate filter sets automatically. Twenty planes with a z-step of 0.8 µm were captured using the Spinning Disk Confocal mode. After acquisition, maximum intensity projections were calculated using the EBImage package in R (Version 4.32.0). Images were then exported, and the various channels were combined in Image J (Version 1.46, National Institutes of Health, Bethesda, MD, USA).

### 4.17. Confocal Imaging and Image Analysis

Plates were imaged using the Zeiss LSM 800 laser scanning microscope and 40×/1.3 NA Plan Apochromat oil immersion lens (BIRU Facility; University of Auckland). Emission and excitation wavelengths for each dye were selected according to the manufacturer’s defaults within the Zeiss Zen software. Images were captured at a resolution of 1437 × 1437 px with two times averaging. Stacks were captured with a z-step of 0.21 µm, with the total number of images selected to capture the full sample thickness. After capture, images were imported to Imaris (Version 9.9.0, Bitplane AG, Zurich, Switzerland). Images were saved in the “lsm” format, and 3D reconstruction and z-stack analysis were performed using Imaris software.

### 4.18. FACS Sorting of NZM7-S

Sorting of the NZM7-S material was conducted by Mr. Stephen Edgar, manager of the flow cytometry facility in the Faculty of Medical and Health Sciences, University of Auckland. NZM7-S material was labelled as described previously with 250 nM CTR, and populations were sorted into 15 mL falcon tubes using a BD FACSAria II SORP cell sorter (BD Biosciences) with a 100 µm nozzle and sheath pressure of 20 psi. The standard settings for the “purity” level of precision were used. Gates in plots of SSC-A vs. SSC-H and FSC-A vs. FSC-H were used to select single particles/cells. Fluorescence of CytoTrack Red was excited with a 640 nm laser, and emission was detected with a 670/30 band pass filter preceded by a 685 long pass dichroic mirror. There were two main populations of particles/cells, judging from the CytoTrack Red fluorescence. The less fluorescent particles were designated “CTR+”. The brighter population was named “CTR++”.

The gating strategy was used to exclude the living melanoma cells (much smaller side scatter population), which were present at low frequency. This was a deliberate step, as it was evident that the NZM7-S barrier disrupting activity was not due to the small number of live melanoma cells present in the NZM7-S material. Immediately after the sorting, which took approximately 40–60 min, the CTR+ and CTR++ material was prepared for addition to the hCMVECs pre-grown on ECIS plates.

### 4.19. Statistical Analysis

RStudio (version 1.1.414, RStudio, Inc., Boston, MA, USA) and vascr (developed by our lab [55,58,67] were used to generate a two-way analysis of variance followed by Tukey’s range test. All probabilities shown are relative to a media only control at the 60 h time point. Normality was confirmed using both visual inspection of the data and the Shapiro–Wilk test of normality. All graphs were generated using GraphPad Prism 7 (La Jolla, CA, USA). The number of independent experiments is detailed in the relevant figure legends.

## 5. Future Perspectives

The next logical step is to understand the prevalence of these vesicular-bodies in the circulation of patients. In order to achieve this, we will need to comprehensively molecularly profile the different vesicle-species produced by the melanoma cells. This will involve extensive proteomic, lipidomic and transcriptomic analyses to reveal the range of species present and markers they express to enable analysis in patient samples. This will also potentially identify molecules that the vesicles use to enter the endothelium and also identify cargo that is transferable or influential. This will be a significant undertaking but will reveal key information for future prognostic, diagnostic and target identification. Key future research needs to determine how to mitigate and prevent the effects of these melanoma-derived vesicles and block their impact on the vascular niches at sites of metastasis. The clinical relevance of these considerations is both obvious and substantial.

## Data Availability

Not applicable as data is contained within the article or supplementary material. Raw data may be provided if requested.

## Supplementary Materials

The following supporting information can be downloaded at: https://www.mdpi.com/article/10.3390/ijms24076082/s1.
