# Supplementary material for: Melanoma Cells Produce Large Vesicular-Bodies That Cause Rapid Disruption of Brain Endothelial Barrier-Integrity and Disassembly of Junctional Proteins
Source: Int J Mol Sci. 2023 Mar 23;24(7):6082. doi: 10.3390/ijms24076082 (PMC10093843; doi:10.3390/ijms24076082)
Supplement: Supplementary file 1 [file ijms-24-06082-s001.zip › ijms-2102638-supplementary.pdf]

Supplemental Figures

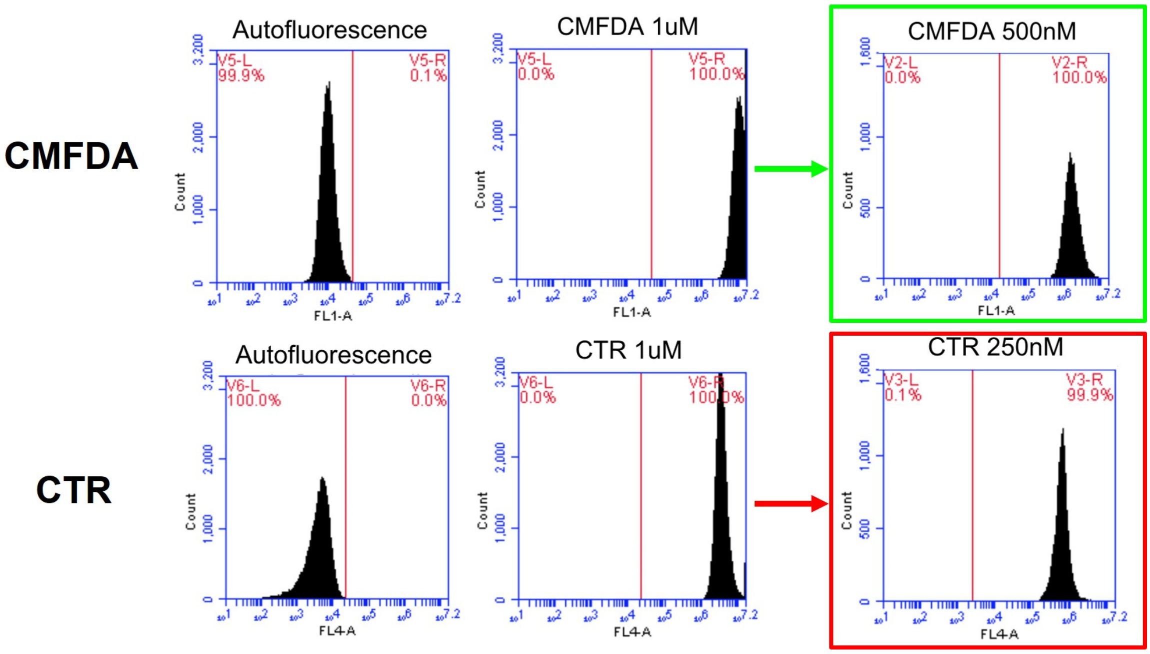

Supplemental Figure 1. Optimisation of cell-tracker stain concentration for detection with flow cytometry. Histograms showing cell-trackers fluorescence; top panel: CellTracker green (CMFDA) and bottom panel: CytoTrack red (CTR) in channels FL1 and FL4 respectively. The boxes from left to right display the autofluorescence of the NZM7 cells, followed by detection at a concentration of 1 µM, deemed too high as the fluorescence was off the scale. The green and red boxes show the optimised concentration for the CMFDA and CTR stains, respectively.

NZM7 cells

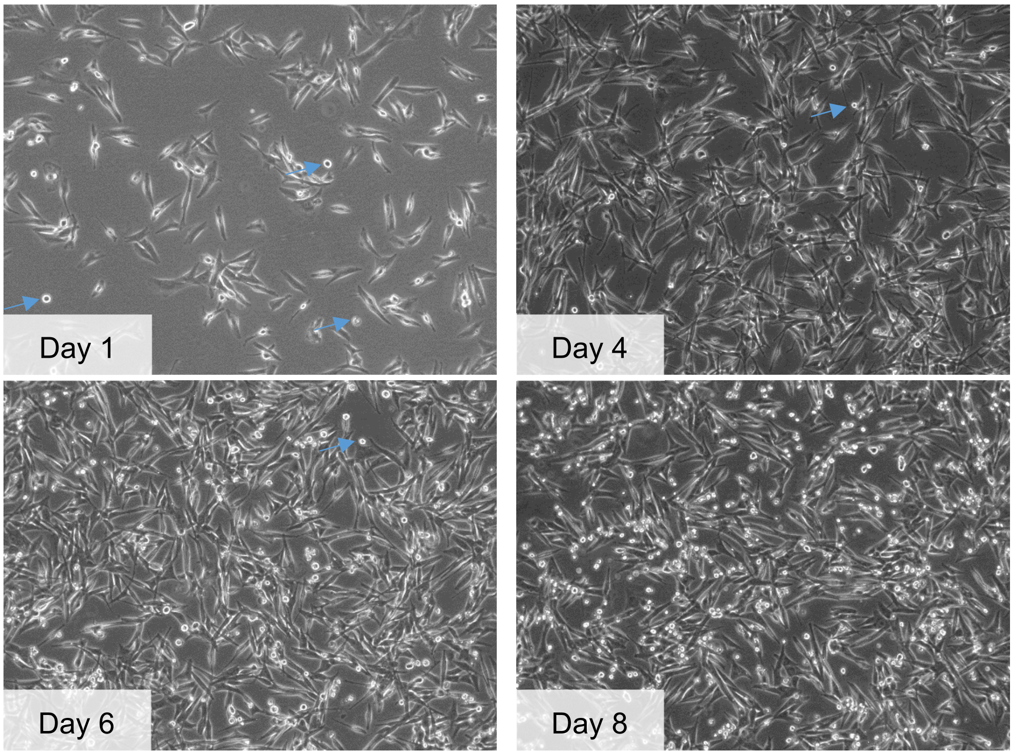

Supplemental Figure 2. Panels show NZM7 cells cultured for 8 days, with phase contrast images acquired on respective days to highlight the presence of the NZM suspension material. The blue arrows highlight examples of the suspension population. The NZM7 cultures produce a considerable amount of this material, which appears phase bright and spherical and appears to be reasonably stable in culture with time.

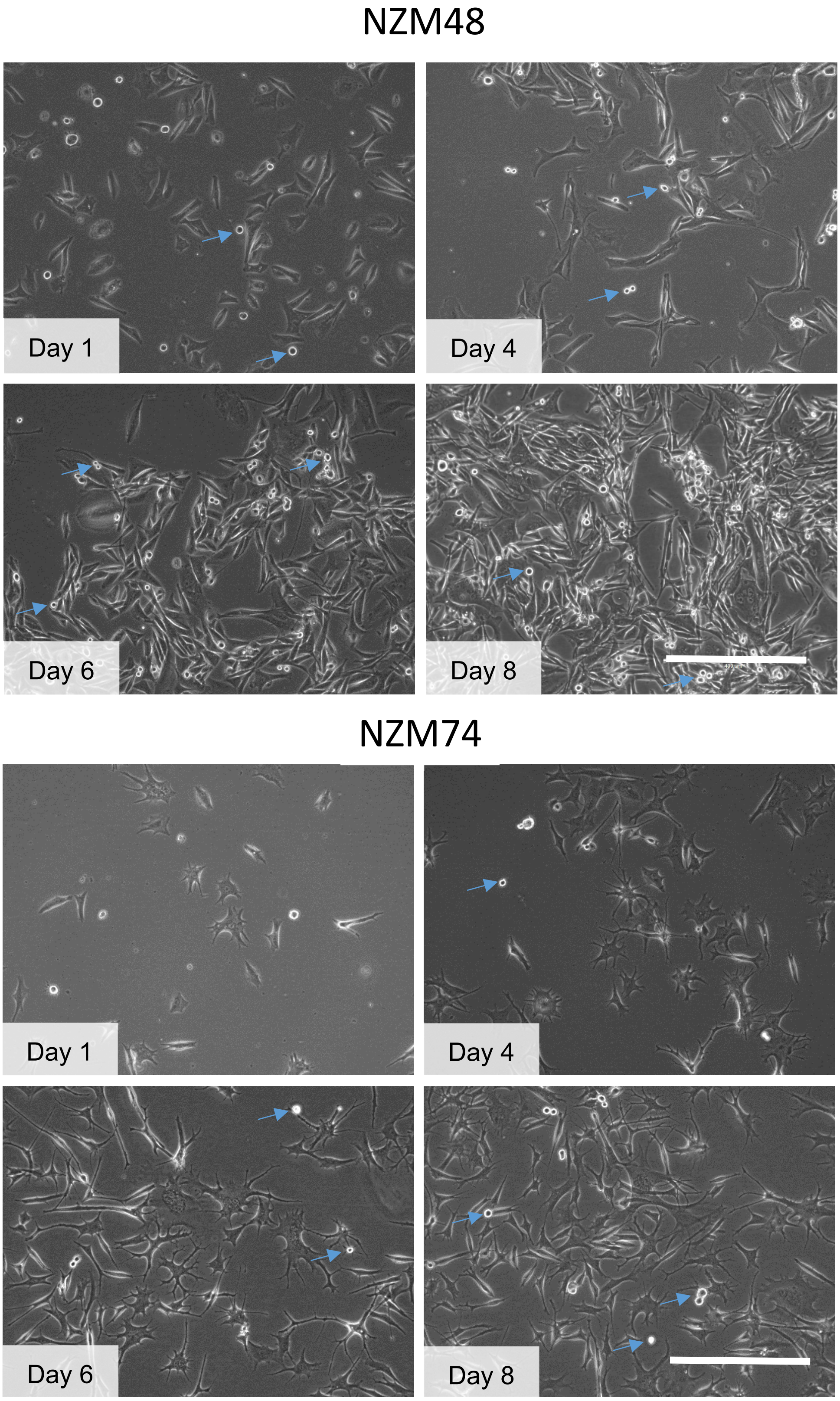

Supplemental Figure 3. Presence of melanoma suspension material in additional melanoma cultures (NZM48 and NZM74). Melanoma cells were seeded at low density and cultured for 8 days. The small blue arrows highlight examples of “suspension material” which does not have the normal adherent phenotype. The large scale bar represent 400 µm.

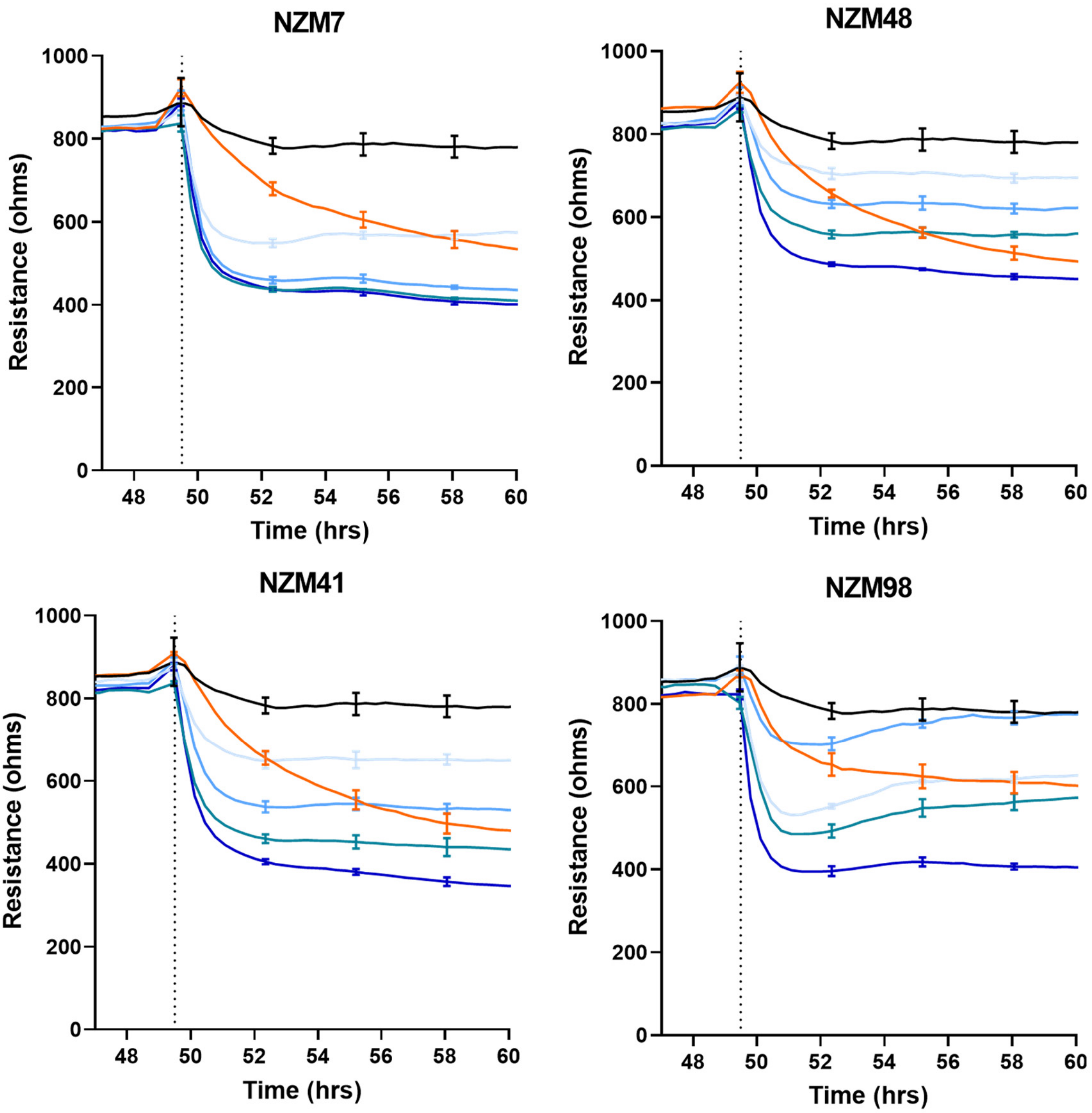

-- Suspension 100%
-- Suspension 50%
-- Suspension 25%
-- Suspension 10%
-- Adherent melanoma 1:1
-- αMEM Control

Cell Line	100%	50%	25%	10%
NZM7	16:1	8:1	4:1	2:1
NZM48	6:1	3:1	1:1	1:2
NZM41	8:1	4:1	2:1	1:1
NZM98	11:1	6:1	3:1	1:1

Supplemental Figure 4. Comparative NZM-**A** and NZM-**S** mediated decrease in cerebral endothelial barrier resistance in 4 different melanoma lines. Graphs show unmodelled resistance (at 4000 Hz) of hCMVECs over time after the addition of the NZM lines. hCMVECs were seeded at 20,000 cells per well. NZM-**adherent** cells were added at the Effector: Target (E:T) ratio of 1:1; 1 NZM cell added to 1 endothelial cell. All NZM-**suspension** material were observed for as long as possible, provided that the adherent cells were viable, and the suspension material was collected on the latest possible day. These were added at 100%, and then diluted to 50%, 25% and 10% to assess any titration effect. Table shows the closest approximate counted titration of each melanoma lines' suspension material. Data show the mean ± SD (n= 3 wells) from 1 experiment only.

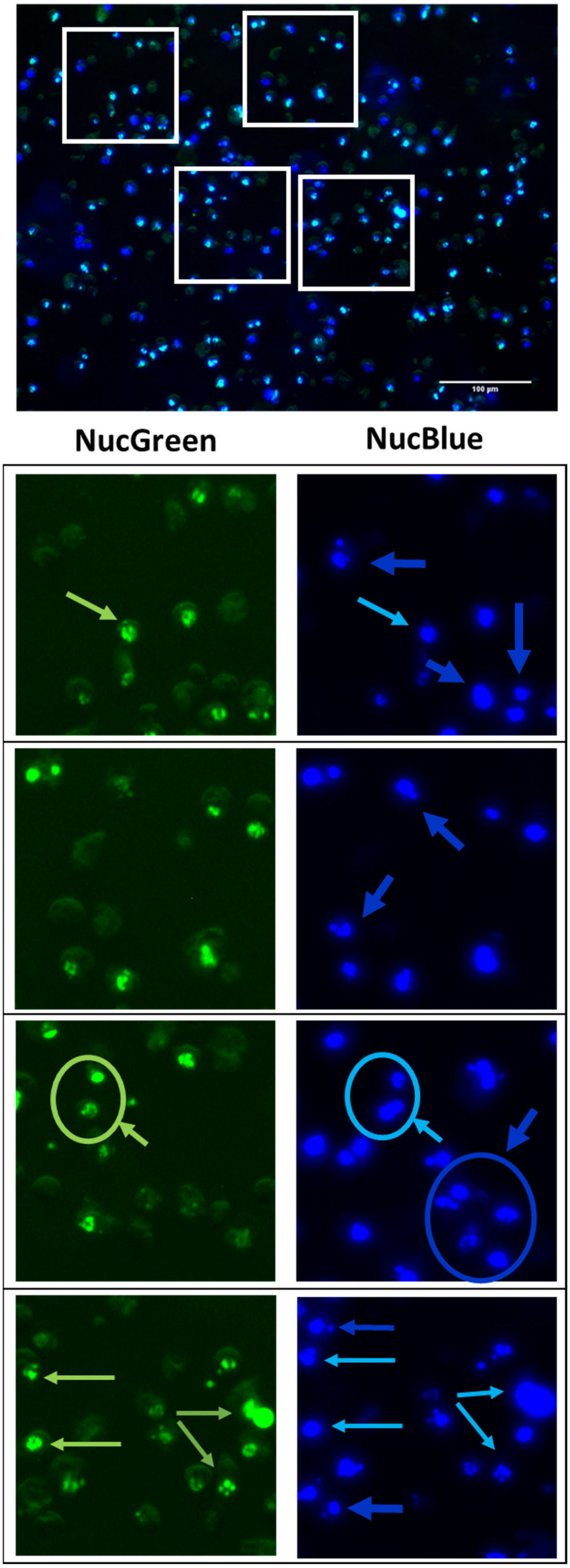

Supplemental Figure 5. NucBlue-live and NucGreen-dead fluorescence stains. Fluorescent imaging of the NZM7-S nuclei using the ReadyProbes™ Blue/Green Viability Imaging Kit (cat# R37609). In practice, the blue/green stains bind DNA where the blue-dye, is membrane-permeant, and the green-dye is membrane-impermeant. The figure shows that some NZM7-S were positive for 'live-cell' nuclear stains (blue arrows), but several were positive for both live and dead nuclear stains as indicated by the teal arrows. This was surprising and suggested that NZM7-S had a nucleus (stained blue), and several – but not all – of these had a compromised membrane (green arrow).

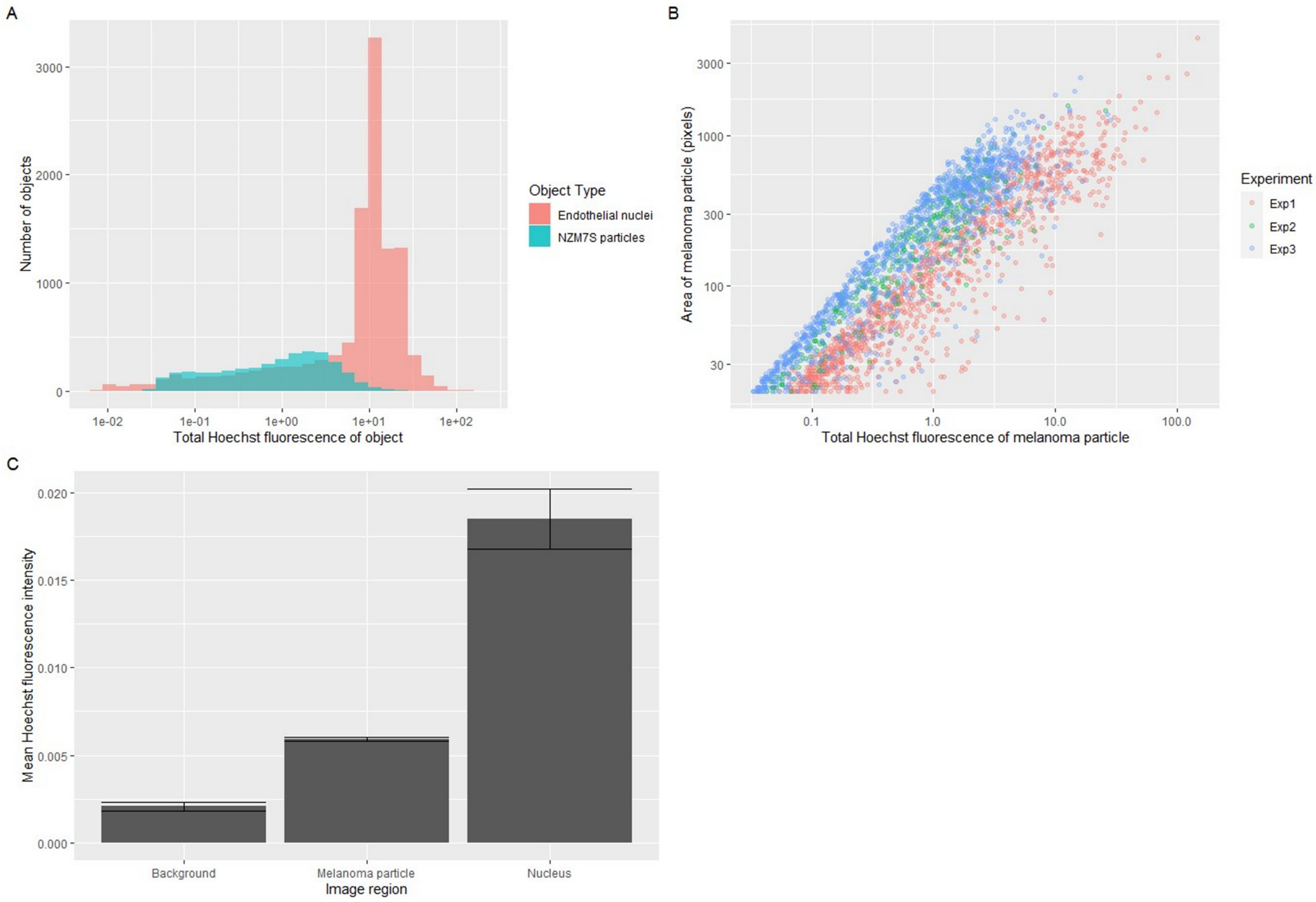

Supplemental Figure 6: Analysis of the Hoechst fluorescence in NZM7-S particles. NZM7-S particles were detected based on their CytoTrack Red (CTR) stain using a local thresholding approach. Melanoma particles were detected between the range of 5-100 pixels, pertaining to sizes 2.5-500 microns in diameter. Endothelial nuclei were detected by applying a local threshold to areas of nuclear stain without CTR co-localization, detecting particles of between 10-40 pixels in diameter, pertaining to sizes 5-20 microns in size. Representative images were visually inspected to ensure the particles were accurately detected. A: shows the total Hoechst intensity within each area identified as endothelial nuclei (teal - Nuclei) or NZM7-S particles (red - Melanoma particles). The intensity of this nuclear stain was variable across the melanoma particles, but distinctly lower than that observed within endothelial nuclei. B: shows across 3 independent experiments that a linear relation is evident between the size of the NZM7-S particles and the total Hoechst fluorescence detected within the melanoma particles. NZM7-S E:T ratio of 2:1 was used for this figure, however all E:T ratios showed a similar trend. C: shows the calculated mean Hoechst fluorescence within all areas identified as either nuclei or melanoma particles. Mean fluorescence intensities were calculated to correct for the differing sizes of these three types of area. This showed the mean Hoechst fluorescence within NZM7-S melanoma particles is substantially greater than that observed in the cytoplasm of hCMVEC cells overall, but acutely less than that occurring in the endothelial nucleus.

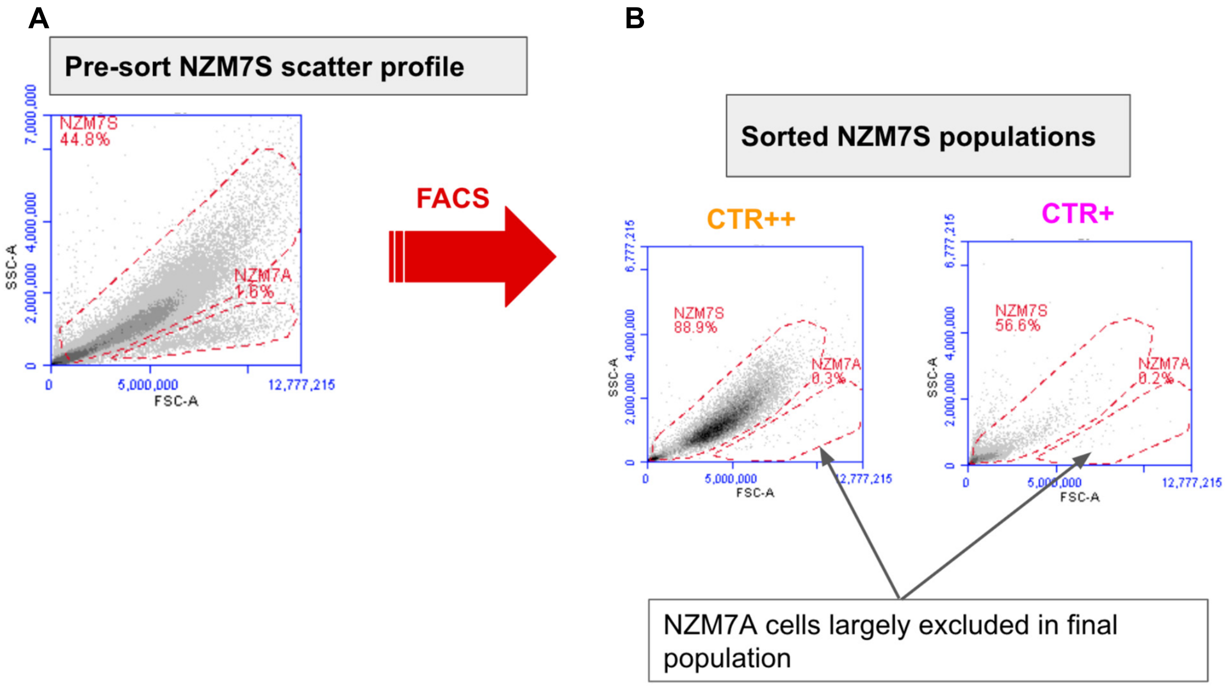

Supplemental Figure 7. Accuri C6 scatter profiles of FACS sorted populations of NZM7-S based on CTR fluorescence intensity. A: shows the scatter profile of the NZM7-S sample before FACS. B: shows the scatter profile of the hyperfluorescent CTR++ and hyperfluorescent CTR+ samples added to the ECIS experiments. Both profiles show the removal of the adherent cell population, seen in the pre-sorted NZM7-S material. All effects of the CTR++ and CTR+ on the endothelial barrier are that of the NZM7-S population alone.

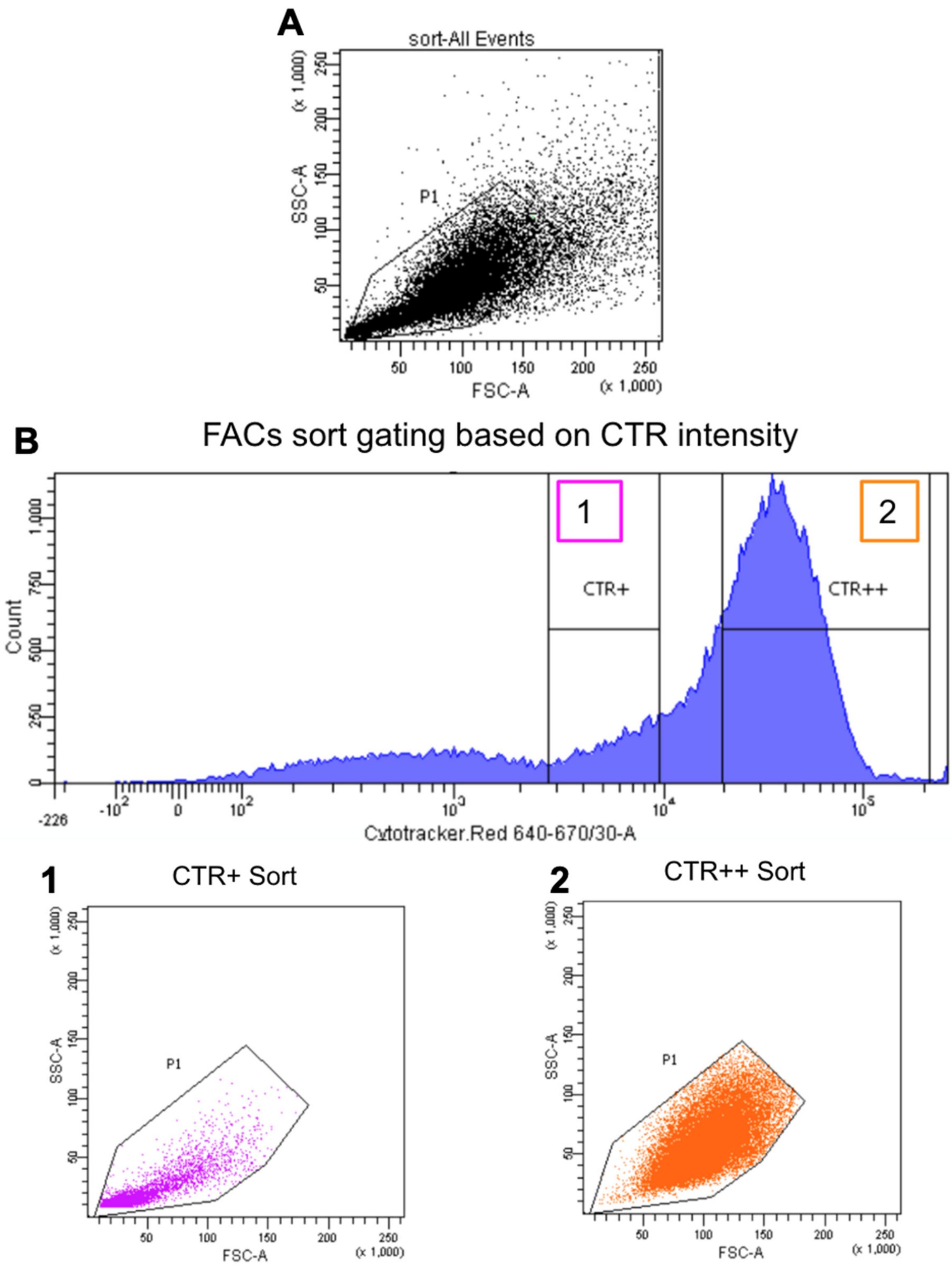

Supplemental Figure 8. FACS Aria II sort data. A: shows gated population of CTR labelled NZM7-S population to be sorted with the BD *FACSAria* flow cytometer. B: shows *FACSAria* histogram depicting the fluorescent intensity of CTR in NZM7-S cells, gated for two different populations. The two populations are 1. Low CTR fluorescence sorted into the CTR+ gate and 2. High CTR fluorescence sorted into the CTR++ gate. 1 and 2 show the two final populations as a result of the FACS gate. Data is representative of 2 independent experiments.
